# Bioactive lipids as biomarkers of adverse reactions associated with apheresis platelet concentrate transfusion

**DOI:** 10.3389/fimmu.2023.1031968

**Published:** 2023-04-17

**Authors:** Anne-Claire Duchez, Sébastien Fauteux-Daniel, Caroline Sut, Theo Ebermeyer, Marco Heestermans, Charles-Antoine Arthaud, Marie-Ange Eyraud, Amélie Prier, Estelle Audoux, Justine Bertrand-Michel, Bernard Payrastre, Olivier Garraud, Eric Boilard, Hind Hamzeh-Cognasse, Fabrice Cognasse

**Affiliations:** ^1^ Etablissement Français du Sang Auvergne-Rhône-Alpes, Saint-Étienne, France; ^2^ SAINBIOSE, INSERM, University of Saint-Etienne, Saint-Étienne, France; ^3^ MetaboHUB-MetaToul, National Infrastructure of Metabolomics and Fluxomics, Toulouse, France; ^4^ I2MC, Université de Toulouse, Inserm, Université Toulouse III – Paul Sabatier (UPS), Toulouse, France; ^5^ INSERM UMR, ToNIC: Toulouse NeuroImaging Centre, Toulouse, France; ^6^ Department of Infectious Diseases and Immunity, Centre de recherche du CHU de Québec, Québec, QC, Canada; ^7^ Université Laval and Centre de recherche ARThrite, Québec, QC, Canada

**Keywords:** inflammation, platelet, transfusion, lipid mediator, adverse reaction (AR)

## Abstract

Platelet concentrate (PC) transfusion seeks to provide haemostasis in patients presenting severe central thrombocytopenia or severe bleeding. PCs may induce adverse reactions (AR) that can occasionally be severe (SAR). PCs contain active biomolecules such as cytokines and lipid mediators. The processing and storage of PCs creates so-called structural and biochemical storage lesions that accumulate when blood products reach their shelf life. We sought to investigate lipid mediators as bioactive molecules of interest during storage and review associations with adverse reactions post-transfusion. To facilitate understanding, we focused on single donor apheresis (SDA) PCs with approximately 31.8% of PCs being delivered in our setting. Indeed, pooled PCs are the most widely transfused products, but the study of a single donor lipid mediator is easier to interpret. We are investigating key lipid mediators involved in AR. Adverse reactions were closely monitored in accordance with current national and regional haemovigilance protocols. Residual PCs were analysed post-transfusion in a series of observations, both with and without severe reactions in recipients. A decrease in the lysophosphatidylcholine species to produce the lysophosphatidic acid species has been observed during storage and in the case of AR. Lysophosphatidic acid increased with primarily platelet-inhibitor lipids. Anti-inflammatory platelet-induced inhibition lipids were weakly expressed in cases of severe adverse reactions. We therefore propose that a decrease in lysophosphatidylcholine and an increase in lysophosphatidic acid can prospectively predict serious adverse transfusion reactions.

## Highlights

- LPA & mostly platelet-inhibitor lipids increased during SDA-PC storage
- LPC & LPA balance could be markers of AR in SDA-PC transfusion

## Introduction

Blood transfusion is used to save lives and promote health through medical and surgical procedures. Platelet concentrate transfusions are used in life-threatening conditions in which patients are exposed to severe thrombocytopenia, especially of central origin, and severe bleeding of varying aetiology (1). Different types of transfusion concentrates are available such as single donor apheresis (SDA) PCs. Although transfusion is usually beneficial to the recipient, adverse reactions may occur (2). They could be immediate or delayed, mild or severe (1). According to haemovigilance registries, PC transfusion is linked more closely with complications than other common blood components. On average, 359/100,000 PCs are associated with severe complications (1, 3, 4). Adverse transfusion reactions (AR) mechanisms are poorly understood. However, when transfusion is associated to ARs, most reactions are inflammatory, present an allergic-type (with no identified allergen or specific IgE), and Febrile non-Haemolytic Transfusion Reactions (FNHTRs) (5). It has been consistently shown that accumulations of Biological Response Modifiers (BRM), produced in non-random profiles, but also mitochondrial DNA and altered lipids are responsible of adverse reaction following a PC transfusion (1, 6). These reactions are induced by biomolecules such as cytokines and lipids as well as storage (time, temperature, etc.). PC processing also plays a significant role in terms of proteomic changes that can escalate into adverse reactions (7), although it did not alter the lipidome (8).

Platelet concentrates comprise platelets - small enucleate cells produced by megakaryocytes. Their main role is to maintain haemostasis but in the last decade, they have been seen to play an important role in immunity and inflammation. Their involvement in inflammatory disease (9–11), cancer (12) and infection is increasingly highlighted. Platelet functions are regulated by surface receptors reliant on the lipid membrane and generation of bioactive lipid mediators (13). Lysophospholipids and fatty acids are released from phospholipids in the plasma membrane by phospholipase. Fatty acids such as arachidonic acid (AA), linoleic acid (LA), docosahexaenoic acid (DHA) and eicosapentaenoic acid (EPA) are metabolised by several enzymes to generate lipid mediators. Depending on their origin, metabolised fatty acids produce pro- or anti-inflammatory lipids, activating or inhibiting lipids, anti or pro-thrombotic lipids such as prostaglandin, leukotrienes and Specialized Pro-*resolving* Mediators (SPM). Platelets generate these various categories alone or in combination with red and white blood cells (14, 15).

We decided to evaluate lipidomes during SDA-PC storage and in ARs in order to assess whether or not lipids are also involved in this pathophysiological process. Indeed, mass spectrometry and ELISA analyses revealed that some lipids such as autotaxin (ATX) products and eicosanoids were significantly involved in AR occurrences post-SDA-PC transfusion. We selected SDA-PC (although it is not the most widely transfused PC in France) over Buffy coat (BC-PC), which is pooled from five PC donors. SDA-PCs induce adverse reactions with a higher frequency compared to BC-PC. The SDA-PC lipidomic data provide a clear-cut analysis, showing the donor effects.

## Methods

### Ethic statement

Single Donor Apheresis - Platelet Concentrates (SDA-PC) were obtained from “Etablissement Français du Sang (EFS) Auvergne-Rhone-Alpes” with 9,206 volunteers enrolled between March 2013 and February 2016 giving their informed consent. The study was approved by EFS’s institutional review board for ethics (DC-2019-3803 & AC-2020-3959) (16). The residual SDA-PCs transfused were collected. Lipidomic analysis was performed on 36 SDA-PC with AR. We randomly selected 20 SDA-PC reported without AR, as controls. More information on the blood donor’s characteristics, the repartition of the sample collection (storage time with the exact number of samples and gender) and the adverse reaction symptom observed are available in **Table 1**. However, we did not have access to clinical data regarding patient histories who receive the transfusion (disease and comorbidity, number of blood product transfusion, especially platelet concentrate, the time of hospitalization, the goal of the transfusion (prophylactic or therapeutic or both)).

**Table 1:** Donor profile, sample and outcome in transfused patient.

	noAR	AR
Individual	20	36
Sex	8 F ± 12 M	12 F ± 24 M
Age	48.93 ± 11.05	44.05 ± 11.83
Body mass index	26.03 ± 2.81	26.29 ± 2.68
Repartition of samples		
storage time	no AR	AR
day 1	3 (1F ± 2M)	3 (1F ± 2M)
day 2	7 (3F ± 4M)	5 (OF ± 5M)
day 3	8 ( 3F ± 5M)	13 (4F ± 9M)
day4	2 (1F ± 1M)	11 (4F ± 7M)
day 5	0	3(2F ± 1M)
Symptoms of adverse reaction		
Chills	–	11
Nausea	–	2
Tachycardia	–	3
Rash	–	8
Fever	–	4
Dyspnea	–	1
Acute lung edema	–	1
Anxiety	–	0
Shock	–	0
Hypertension	–	0
Bradycardia	–	0
Oligo-anuria	–	0
Hemorrhagic syndrome-		0
Hypotension	–	0

### Sample preparation

SDA-PCs were collected as described above (8, 17). Briefly, blood was collected on ACD-A using Trima, a continuous-flow cell separator (Gambro BCT, Lakewood, CO, USA). The SDA-PCs was automatically resuspended in 35% autologous donor plasma and 65% platelet additive solution (PAS-D, Intersol, Fenwal, La Châtre, France; or PAS-E, SSP+, MacoPharma, Mouveaux, France) and stored at 22 ± 2°C with gentle rotation and shaking (60 rpm) for a maximum of 5 days (after collection was completed) before being issued for transfusion.

PCs supernatants were collected after centrifugation (402 × g; 10 min) to remove platelets, aliquoted and frozen at −80˚C until further use for mass spectrometry and ELISA analysis.

### Mass spectrometry

Liquid chromatography tandem mass spectrometry (LC-MS/MS) analysis was performed by the MetaToul-Lipidomic MetaboHUB Core Facility, France. More information is available in the **Supplementary Information**.

### ELISA

SDA-PCs with/without AR were analysed with human ENPP-2/Autotaxin Duo set ELISA (Bio-Techne SAS, Noyal Châtillon sur Seiche, France), as per the company protocol.

Soluble CD62P was quantified with the Milliplex Map Human cardiovascular disease magnetic beab panel 2 (Merck-Millipore), according to the manufacturer’s protocol.

### Principal component analysis

SIMCA 17 software (Umetrics, San Jose, CA) was used to analyse the mean centre and unit variance scaling of lipid mediators detected, SDA-PC with or without AR as described above (18).

Data from the lipidomic analysis (multiple samples for AR and AR storage times) were subjected to principal component analysis (PCA). Each point represents the mean of AR or AR lipid, taking storage times into account. The score plot shows the clustering among observations. The closest lipid, presents greater similarity in the matrix.

### BioPan

A web-based tool was used to assess lipid pathways in order to ensure the non-biased analysis of lipidomic data (BioPAN) (19).

### Statistical analysis

Multiple comparisons were performed by analysis of variance (one way or 2-way-ANOVA, with Sidak test or with Tukey’s multiple comparisons test, and Mann Whitney test. P-values of 0.05 and lower are considered statistically significant (* p < 0.05, ** p < 0.01, ***p < 0.001 and **** p < 0.0001). Statistical analysis and Pearson correlation was carried out using GraphPad version 6 (GraphPad Software, La Jolla California USA).

## Results

To validate our hypothesis on lipid mediator involvement in adverse reaction following a PC transfusion, single donor apheresis platelet concentrates (SDA-PC) were collected from different female and male donors with different ages (22 to 65 years, **Table 1**). After the transfusion of these SDA-PC, donors were classified according to whether they generated adverse reactions (**Supplementary Figure 1A**). Most of PC are transfused a short time after their preparation (until 2 days). For this reason, we decided to merge the lipidomic data of day1 and day2 storage before transfusion, and to merge the data from 3, 4 and 5 day of storage. We performed a targeted lipidomic analysis on the most prominent lysophospholipids and eicosanoids (**Supplementary Table 1**). These lipid mediators are produced by platelets or are involved in their stimulation. We assessed their expression in SDA-PC supernatants (after platelet removal), during storage, in situations with and without an AR. The bioactive lipids measured were free or associated with extracellular vesicle membranes (EV) (14, 20).

In this study, most of the AR symptoms observed were chill, rash and fever. These symptoms are classified as mild rather than severe ARs (**Table 1** and **Supplementary Figure 1A**). Most of the SDA-PCs that induced ARs were derived from normal weight and overweight blood donors, based on BMI calculations (**Supplementary Figure 1B**). Moreover, ARs appeared mostly after the transfusion of SDA-PCs stored for 3-4 days (**Supplementary Figure 1C**). Interestingly, there is a correlation between parameters such as BMI, donor age and PC storage time with adverse reaction symptoms (**Supplementary Figure 1D**). However, our study aimed to decipher the impact of lysophospholipids and lipid mediators along the storage and on the transfusion outcome.

### Lysophosphatidylcholine content, in single donor platelet concentrate, is modulated during storage and is decreased in the event of an adverse reaction

**Figure 1:**
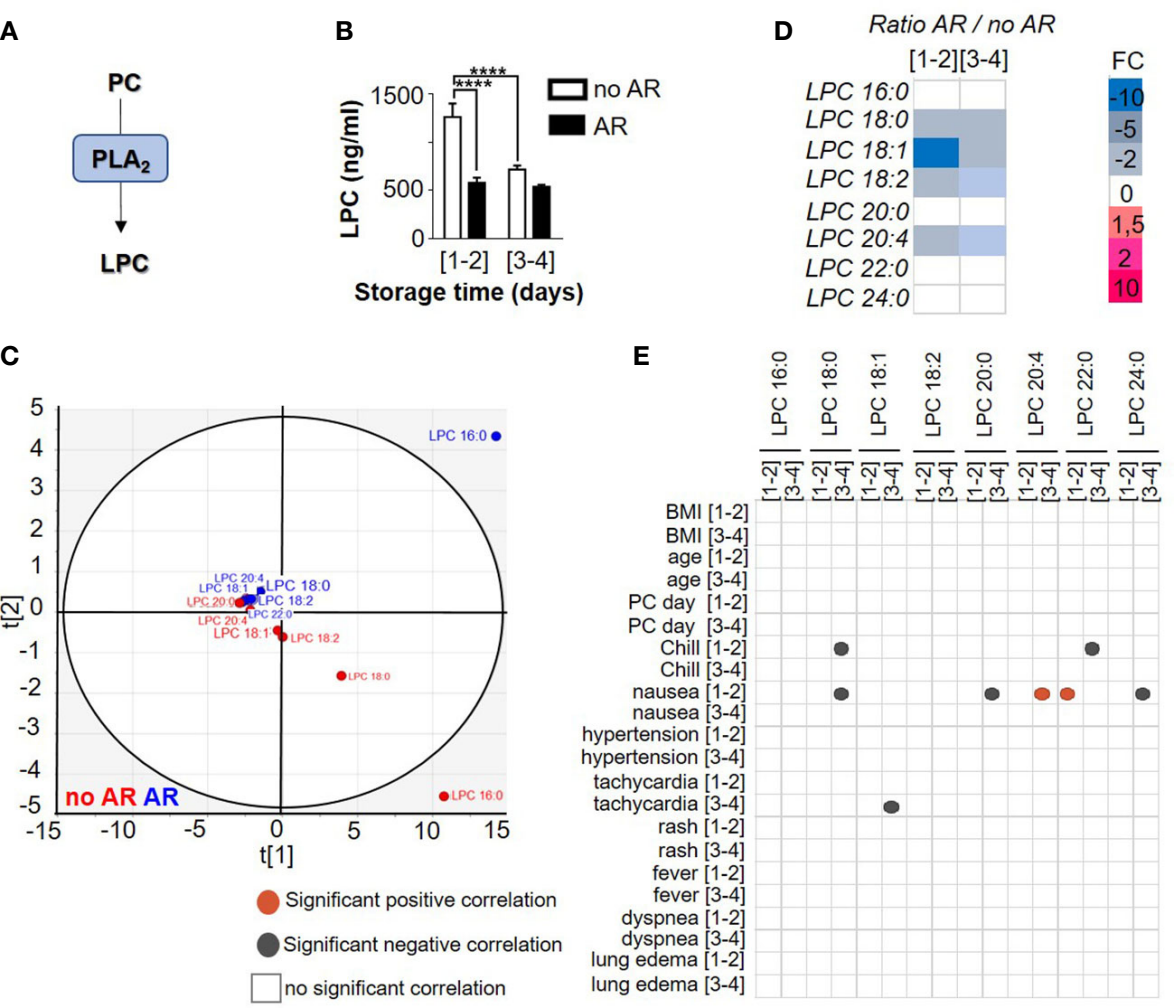
Comparison of lysophosphatidylcholine profile between Single Donor Apheresis platelet concentrate with or without adverse reaction. **(A)** Flowchart illustrating the generation of LPC. **(B)** LPC generation in Single Donor Apheresis platelet concentrates (SDA-PC) with or without adverse reaction (AR). The white bar represents mean ± SEM of LPC expression without AR occurrence and the black bar denotes mean ± SEM of LPC expression with AR occurrence. (to refer to n patient, see **Table 1**). Statistical analysis performed with 2-way ANOVA with Tukey’s multiple comparisons test, p=0.0004, ****p<0.0001. **(C)** Two-dimensional score plot of a principal component analysis of LPC species from SDA-PC without occurrence of AR (red) and from SDA-PC with occurrence of AR (blue) taking all storage times into account. **(D)** Heat map represents fold change in mean LPC species expression between SDA-PC with occurrence of AR and from SDA-PC without occurrence of AR for the various storage times reported on the heat map. **(E)** Correlation matricx between LPC species and adverse reaction symptoms are built with a Pearson correlation. Grey dots represent a negative correlation and red dots a positive correlation, with the level of significance being p <0.05.

The presence of lysophosphatidylcholine (LPC) in PCs has been highlighted in several studies and is generated through PLA_2_ activity (**Figure 1A**). However, no information regarding LPC expressions during storage or AR links has come to light. In this study, LPC expressions (1262,97 ng/ml ± 140,68 for [1-2] and 718, 64 ng/ml ± 41,23 for [3-4], 2-way ANOVA with Tukey’s multiple comparisons test, *p=* 0.0004) detected in transfused PC from different time of storage ([1-2] or [3-4] days) significantly decreased during storage and in the presence of AR (579, 94 ng/ml ± 53,06 for [1-2] and 538,22 ng/ml ± 20,11 for [3-4]) (**Figure 1B**). Interestingly, LPC species clustered according to AR status using a principal component analysis (**Figure 1C**). Indeed, LPC species detected in SDA-PC with AR clustered whereas LPC species from SDA-PC without AR also clustered. These data showed a difference of expression of LPC species during AR. Some LPCs such as LPC 16:0, 18:0, 18:1, 18:2 and 20:4 decreased significantly when the AR was linked to storage. Others (LPC 18:0, 18:1, 18:2 and 20:4) decreased significantly between SDA-PCs that induced AR and SDA-PC without the occurrence of ARs. In contrast, the expression of some LPC species increased significantly in SDA-PC, thereby inducing ARs compared to SDA-PC without ARs. This applied in the case of LPC 20:0, 22:0 and 24:0 (**Figure 1D** and **Supplementary Figure 2A**). LPC species differ in terms of numbers, but some are more prominent during storage such as LPC 16:0, which is also the most abundant in AR cases (**Figure 1D** and **Supplementary Figure 2B**). Interestingly, Pearson correlation matrix between AR symptoms and LPC species concentration along storage showed AR symptoms (**Figure 1E**). Indeed, LPC 22:0 from PC stored [1-2] days and transfused, are positively correlated with nausea symptoms in patient that were transfused with PC stored 1-2 days. LPC 18:1 from [3-4] days of storage is negatively correlated with tachycardia symptoms observed in patients transfused with PC stored 3-4 days. LPC 22:0 and LPC 18:1 could be considered as predictive markers for nausea and tachycardia symptoms observed in patients transfused with PC. However, lysophospholipid expression association with different symptom of AR is not the only factor of AR.

### Autotaxin and their products in single donor platelet concentrate during storage and in the event of an adverse reaction

**Figure 2:**
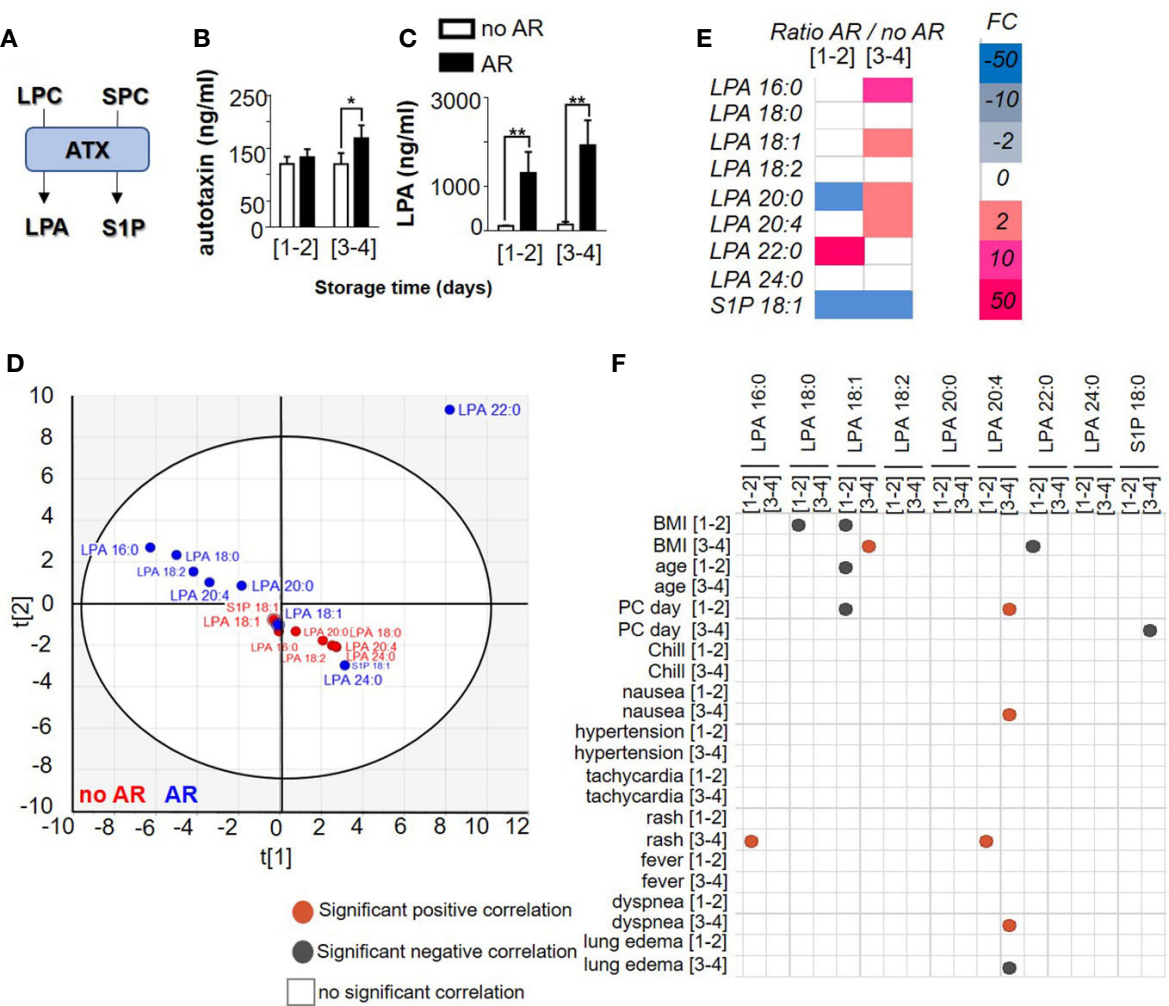
Comparison of autotaxin product profile between Single Donor Apheresis platelet concentrate with or without an adverse reaction. **(A)** Flowchart illustrating the generation of lysophosphatidic acid (LPA) and sphingosine 1 phosphate (S1P) through ATX activities. **(B)** ATX expression in SDA-PC with or without AR occurrence -during storage (mean ± SEM). Statistical analysis performed with Mann Whitney test *p<0.05. **(C)** LPA generation in SDA-PC with or without AR occurrence during storage. The white bar represents SDA-PC without AR occurrence, and the black bar denotes SDA-PC with AR occurrence. (mean ± SEM; to refer to n patient, see **Table 1**). Statistical analysis performed with Mann Whitney **p<0.0001. **(D)** Two-dimensional score plot of a principal component analysis of LPA species and S1P from SDA-PC without occurrence of AR (red) and from SDA-PC with occurrence of AR (blue) taking all storage times into account. **(E)** Heat map represents fold change in mean LPA species and S1P expression between SDA-PC with occurrence of AR and from SDA-PC without occurrence of AR for the various storage times reported on the heat map. **(F)** Correlation matrix between ATX products and adverse reaction symptoms are built with a Pearson correlation. Grey dots represent a negative correlation and red dots a positive correlation, with the level of significance being p <0.05.

Autotaxin (ATX) is a phospholipase D, able to catalyse the hydrolysis of LPC in lysophosphatidic acid (LPA) and to catalyse sphingosylphosphorylcholine (SPC) in Sphingosine-1-phosphate (S1P) (**Figure 2A**). Platelets are the major source of ATX in the blood circulation. Knowing that ATX expression increases during inflammation (21), we measured ATX expression during storage of PC and in the event of AR. We observed stable expression of ATX during storage (120,11 ng/ml ± 13,77 for [1-2] and 119,6 ng/ml ± 21,06 for [3-4] days) but increased in the event of an AR (132,84 ng/ml ± 15,29 for [1-2] and 168,96 ng/ml ± 24,76 for [3-4], Mann Whitney test, *p=* 0.0152) (**Figure 2B** and **Supplementary Figure 3A**). These observations prompted us to investigate LPA and S1P expression during storage and in the event of an AR. We noted that ATX expression increased in case of AR with long storage of PC (**Figure 2B**) whereas LPC (**Figure 1B**) and S1P expression (**Supplementary Figure 3A**) decreased during storage and in the occurrence of AR. LPA increased during storage and when an AR occurred (without AR: 104.44 ng/ml ± 12.56 for [1-2] and 205.30 ng/ml ± 67.06 for [3-4] and with AR: 1295.6 ng/ml ± 475.13 for [1-2] and 1925.77 ng/ml ± 561.22 for [3-4]), Mann Whitney, *p<*0.0001) (**Figure 2C** and **Supplementary Figure 3A**). Otherwise, LPA species in SDA-PCs not linked to ARs clustered in comparison with AR-related SDA-PCs (**Figure 2D**), which suggests that LPA species modulation could be a cause of AR. Indeed, the expression pattern of LPA species changed during storage and also between SDA-PCs with and without ARs (**Figure 2E** and **Supplementary Figure 3A**). Interestingly, Pearson correlation matrix between AR symptoms and LPA species and S1P concentration along storage showed positive and negative correlation, respectively (**Figure 2F**). Indeed, LPA 20:4 expression from PC stored 3-4 days and transfused, positively correlates with nausea and dyspnea symptoms in patient transfused with PC stored 3-4 days. Moreover, LPA 18:1 from 1-2 and 3-4 days of storage correlates with the BMI and the age of the donor. LPA 20:4 from PC stored 3-4 days negatively correlates with lung edema symptoms in patients who received a PC stored 3-4 days.

We then considered a possible correlation between ATX products and LPC species. We initially compared the species without taking in account the storage time. We noted a correlation between some S1P, LPA and LPC species in SDA-PCs without AR (**Supplementary Figure 4B**) or with AR (**Supplementary Figure 4C**) and between SDA-PCs with or without AR (**Supplementary Figure 4D**). We did not observe correlations between LPA 16:0 and LPC 16:0 in PC involved or not in AR (**Supplementary Figures 4B, C**). However, several correlations both positive and negative were observed between different species of LPA, LPC and S1P in SDA-PC without AR, with AR or in comparison between AR and no AR (respectively **Supplementary Figure 4B–D**).

**Figure 3:**
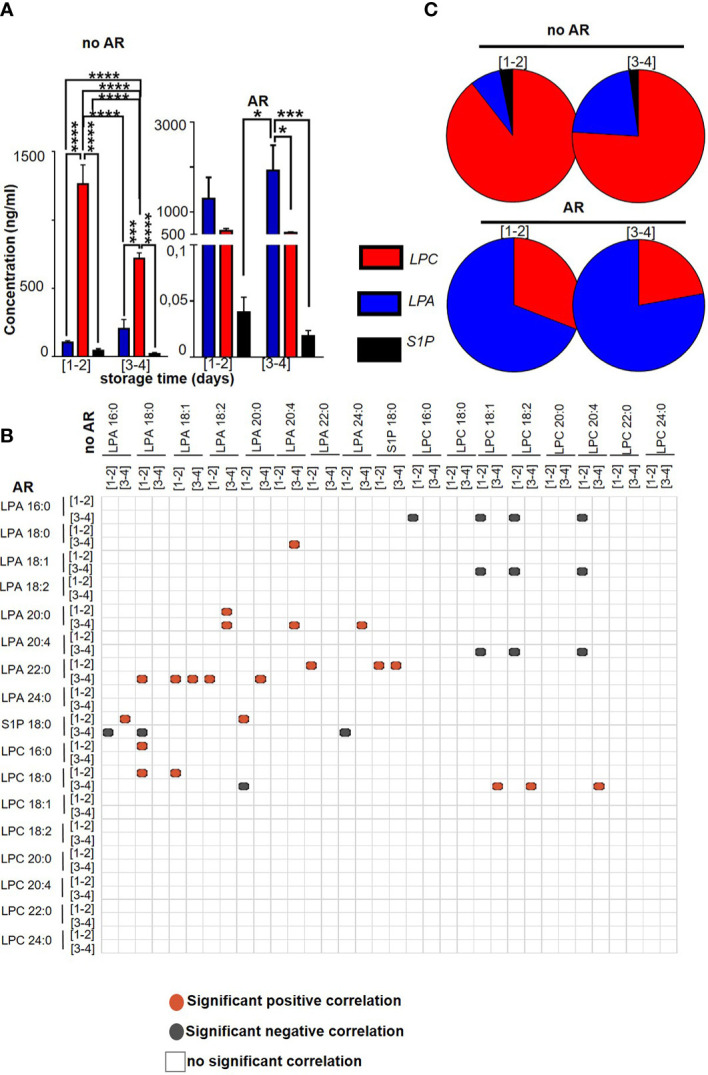
Comparison between LPC, LPA and S1P species contained in Single Donor Apheresis during storage with or without an adverse reaction. **(A)** Graph bar represents the mean ± SEM of the concentration of total LPC (red bar), LPA (blue bar) and S1P (black bar) in SDA-PC without occurrence of AR (no AR, on the left) and with occurrence of AR (on the right). (To refer to n patient, see **Table 1**). Statistical analysis performed with 2-way ANOVA *p<0.05; ***p<0.0005, ****p<0.0001. **(B)** Correlation matrix between expressions of LPA, LPC and S1P in SDA-PC with and without AR. Red dots correspond to significant (p<0.05) and positive correlation with Pearson correlation while the grey/blue dots correspond to significant (p<0.05) and negative correlation with Pearson correlation. The white box corresponds to insignificant correlation. **(C)** The pie chart represents the proportion based on the concentration in ng/ml of LPC, LPA and S1P expression during storage of SDA-PC with or without AR.

We wondered whether storage time was involved in the generation/correlation between LPA, LPC species and S1P depending on the occurrence of AR. Interestingly, combined total LPC species (1262.97 ng/ml ± 140.68 for [1-2] and 718.64 ng/ml ± 41.23 for [3-4]) are by far more prevalent compared to LPA species (104.44 ng/ml ± 12.56 for [1-2] and 205.30 ng/ml ± 67.06 for [3-4]) and S1P (44.96 ng/ml ± 13.39 for [1-2] and 21.82 ng/ml ± 6.45 for [3-4]) in no AR SDA-PC, 2-way ANOVA with Tukey’s multiple comparisons test for no AR *p<*0.0001 for AR, *p=*0.6374; 2-way ANOVA with Sidak’s multiple comparisons test between no AR and AR, *p=*0,7529) (**Figures 3A**, **B**). However, in SDA-PCs inducing an AR, the LPA species (1295.6 ng/ml ± 475.13 for [1-2] and 1925.77 ng/ml ± 561.22 for [3-4]) were more prevalent than LPC (579.94 ng/ml ± 53,06 for [1-2] and 538.22 ng/ml ± 20.11 for [3-4]) and S1P (0.04 ng/ml ± 0.013 for [1-2] and 0.019 ng/ml ± 0.005 for [3-4]) (**Figures 3A**, **C**). Moreover, platelet activation in these SDA-PC was evaluated by measuring soluble P-selectin (sCD62P). We observed an increase of sCD62P in AR as compared to no AR (**Supplementary Figure 5A**), and the expression of sCD62P was positively correlated with S1P 18:1 and LPC 18:2 (**Supplementary Figures 5B, C**). This metabolite of LPA and S1P from LPC thus could be used as an SDA-PC markers to measure before a PC transfusion, in order to prevent AR.

### Eicosanoid content in single donor platelet concentrates during storage and in the event of an adverse reaction

**Figure 4:**
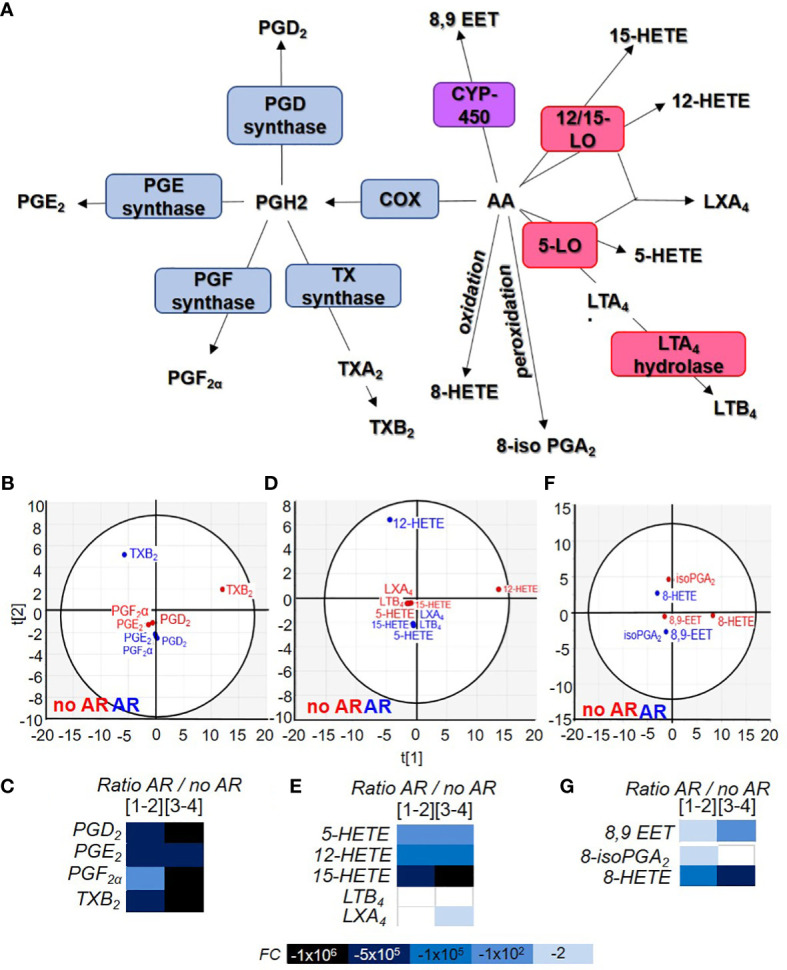
Comparison of acid arachidonic metabolite contained in Single Donor Apheresis during storage and in the event of an adverse reactions. **(A)** Flowchart illustrating the generation of eicosanoids from arachidonic acid (AA) through cyclooxygenase (COX), prostaglandin (PG) synthase, thromboxane (TX) synthase, lipoxygenase (LO) and cytochrome P450 (CYP-450). **(B, D, F)** Two-dimensional score plot of a principal component analysis of COX products from AA **(B)**, LO products from AA **(D)** and CYP-450/oxidation/peroxidation of AA **(F)** in SDA-PC without occurrence of AR (red) and with occurrence of AR (blue), taking all storage times into account. **(C, E, G)** Heat map represents fold change in mean eicosanoid (COX products C), (LO products E) and other products **(G)** expression between SDA-PC with occurrence of AR and from SDA-PC without occurrence of AR for different storage times reported on the heat map. Prostaglandin D_2_ (PGD_2_), prostaglandin E_2_ (PGE_2_), prostaglandin F_2_α (PGF_2_α), thromboxane B_2_ (TXB_2_), 8iso-prostaglandin A_2_ (8iso-PGA_2_), prostaglandin A_1_ (PGA_1_),8-hydroxyeicosatetraenoic acid (8-HETE), 12-hydroxyeicosatetraenoic acid (12-HETE), 15-hydroxyeicosatetraenoic acid (15-HETE), 5-hydroxyeicosatetraenoic acid (5-HETE), leukotriene B_4_ (LTB_4_), lipoxin A_4_ (LXA_4_), 8,9-epoxyeicosatrienoic acid (8,9-EET).

**Figure 5:**
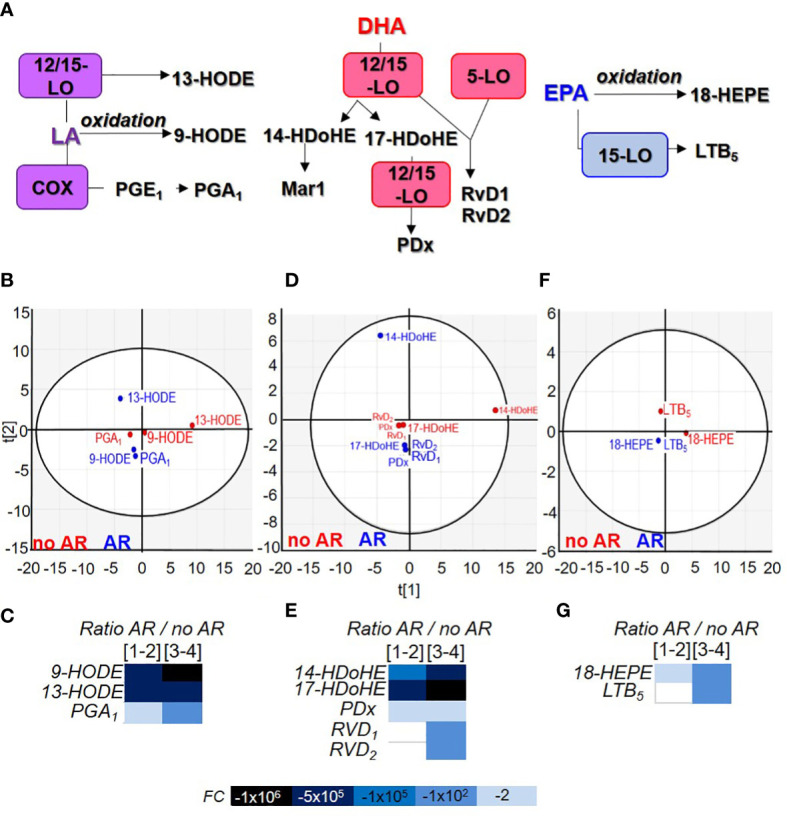
Comparison of linoleic acid, docosahexaenoic acid and eicosapentaenoic acid metabolite contained in Single Donor Apharesis during storage and in the event of adverse reactions. **(A)** Flowchart illustrating the generation of eicosanoids from linoleic acid (LA), docosahexaenoic acid (DHA) and eicosapentaenoic acid (EPA). **(B, D, F)** Two-dimensional score plot of a principal component analysis of LA products **(B)**, DHA products **(D)** and EPA products **(F)** in SDA-PC without occurrence of AR (red) and with occurrence of AR (blue), taking all storage times into account. **(C, E, G)** Heat map represents fold change in mean lipid derived from LA **(C)**, DHA **(E)** and EPA **(G)** between SDA-PC with occurrence of AR and from SDA-PC without occurrence of AR for different storage times reported on the heat map. Leukotriene B_5_ (LTB_5_), resolvin D_1_ (RvD_1_), resolvin D_2_ (RvD_2_), protectin Dx (PDx), 9-hydroxyoctadecadienoic acid (9-HODE), 13-hydroxyoctadecadienoic acid (13-HODE), 14-hydroxydocosahexaenoic acid (14-HDoHE), 17-hydroxydocosahexaenoic acid (17-HDoHE), 18-hydroxyeicosapentaenoic acid (18-HEPE).

Lysophospholipids are not the only bioactive lipids in circulation (22). The various eicosanoids we measured are divided according to the fatty acid of origin such as arachidonic acid (AA), linoleic acid (LA), docosahexaenoic acid (DHA) and eicosapentaenoic acid (EPA). Lipids could be metabolised by cyclooxygenase (COX), lipoxygenase (LO), cytochrome P450 (CYP-450) or peroxided and oxidised (**Figures 4A**, **5A**).

### Arachidonic acid-derived lipid mediator’s expression is modulated during storage and in the event of an adverse reaction

Arachidonic acid is an omega 3 and omega 6 essential fatty acid. It is a precursor for all prostaglandins, thromboxanes, and leukotrienes (**Figure 4A**). Principal component analysis shows different clusters based on AA’s COX products from SDA-PC with or without the occurrence of AR. TXB_2_ is distant compared to others (**Figure 4B**). TXB_2_ was significantly more elevated in no AR-related samples compared to controls and as a result of storage (**Supplementary Figure 6A**). PGD_2_, PGE_2_ and TXB_2_ were also significantly lower in AR-related samples (**Figure 4C** and **Supplementary Figure 6A**). Indeed, the ratio between prostaglandin from SDA-PC without AR and SDA-PC with AR revealed a substantial decrease in SDA-PC prostaglandin expression that led to AR (**Figure 4C**).

AA-derived leukotrienes and di-enes are mostly known as pro-inflammatory lipids. Leukocytes such as neutrophils and monocytes expressed 5-lipoxygenase (5-LO) and 15-LO enzymes to generate 5-HETE and 15-HETE. Reticulocytes express 15-LO. However, platelets do not express 5-LO and 15-LO but LTC_4_ synthase. It is interesting to note the increase in these products in SDA-PCs despite the fact that they are by definition leucocyte- and reticulocyte-depleted. The link to environmental contamination cannot, however, be ruled out (**Supplementary Figure 6B**). Principal component analysis showed a cluster of this bioactive lipid based on SDA-PC with or without the occurrence of AR (**Figure 4D**). Interestingly, in AR-related samples, all LO products were either missing or their concentrations were lower than in non-AR-related samples (**Figure 4E** and **Supplementary Figure 6B**). Finally, LTB_4_ and LXA_4_ expression was decreased during storage and in samples leading to an AR, respectively.

Finally, AA could be metabolised by cytochrome P450 (CYP450) present in human platelets (23) to generate 8,9-EET during concomitant activity with PLA_2_ (24). These lipids did not cluster according to group (belonging to SDA-PC with or without AR) (**Figure 4F**). Its expression increased during storage and decreased in AR-related samples (**Figure 4G** and **Supplementary Figure 6C**). AA could also be transformed without an enzyme into 8iso-PGA_2_ and 8-HETE). Their expressions are increased during storage but strongly decrease in AR-related SDA-PCs (**Figure 4G** and **Supplementary Figure 6D**).

### Linoleic acid-derived lipid mediator’s expression is constant during storage but modulated in the event of an adverse reaction

Linoleic acid (LA) is an essential omega 6 fatty acid present in copious quantities in food and especially in vegetable oils, meats and nuts. It can be metabolised into different bioactive lipids (**Figure 5A**). LA metabolites clustered according to the AR classification whereas 13-HODE differentiated itself depending on whether or not the SDA-PC lead to an AR (**Figure 5B**). Their expression was not modulated during storage but strongly decreased in SDA-PC which induced AR (**Figure 5C** and **Supplementary Figure 6E**).

### Docosahexaenoic and eicosapentaenoic acid-derived lipid mediators’ expression is modulated during storage and decrease in the event of an adverse reaction

DHA is a long chain omega 3 polyunsaturated fatty acid. It is found in fish and algal oils. It is stored mainly in the brain (accounts for 40% of the total polyunsaturated fatty acid of the brain). It is produced by α-linoleic acid. DHA can also be released from the plasma membrane by PLA_2_ and metabolised in specialised pro-resolving mediators (SPM) such as protectins, resolvins and maresins (**Figure 5A**). They are all clustered according to the AR group except 14-HDoHE (**Figure 5D**). 17-HDoHE and RvD_1_ expression increased significantly during storage while 14-HDoHE, PDx and RvD_2_ were not modulated. All DHA product expression decreased in the event of AR (**Figure 5E** and **Supplementary Figure 6F**).

EPA is an omega-3 fatty acid mostly found in marine organisms. EPA products in SDA-PC were clustered according to the AR group (**Figure 5F**) and were not significantly modulated during storage. However, 18-HEPE expression decreased significantly in SDA-PC thereby resulting in AR compared to SDA-PC without AR (**Figure 5G** and **Supplementary Figure 6G**).

The principal metabolites in SDA-PCs are 12-HETE>13-HODE>14-HDoHE. Their expressions were modulated during storage and in conditions leading to an AR (**Supplementary Figure 7H**). 12-HETE has a higher expression in SDA-PC which led to AR with symptoms of rash (**Supplementary Figure 7A**) and, inversely, we observed a lower expression of 12-HETE in SDA-PC resulting from an AR with chills compared to SDA-PC with AR symptoms excluding rash and chills, respectively (**Supplementary Figure 7A**). However, 12-HETE cannot be considered alone to induce an AR or specific AR symptoms. Although no significant difference was observed, there was evidence of some correlation between eicosanoid expressions and AR symptoms (**Supplementary Figure 7B**). However, we did not observe any correlation between platelet activation and lipid mediators, in the presence or absence of AR (**Supplementary Figures 5B, C**). We queried whether there was a correlation between these lipids and the occurrence of AR, taking the storage time into account. Age and BMI did not correlate with any eicosanoids. However, PGD_2_ expression on day 1-2 correlated with the “age” of the PC, for example. In summary, we observed several correlation between oxylipin and AR symptom, however, other circulating molecules such as cytokine, in combination with some oxylipin are associated with AR symptoms.

### Bioinformatic analysis reveal a prominent pathway during the storage of single donor apharesis in the event of an adverse reaction

Various LPC, LPA, eicosanoid species and S1P were measured in our study. Lysophospholipids are mostly expressed in SDA-PC, with or without occurrence of AR. In order to assess the impact of lysophospholipid and/or eicosanoid on the occurrence of AR, we performed a bioinformatic analysis of all data using BioPan software. Bioinformatics Methodology For Pathway Analysis (BioPan) is a bioinformatic tool to investigate lipidomic pathway with our set of data. The pathways are built from literature. The analysis highlighted the change at the lipid subclasses or lipid molecule levels.

**Figure 6:**
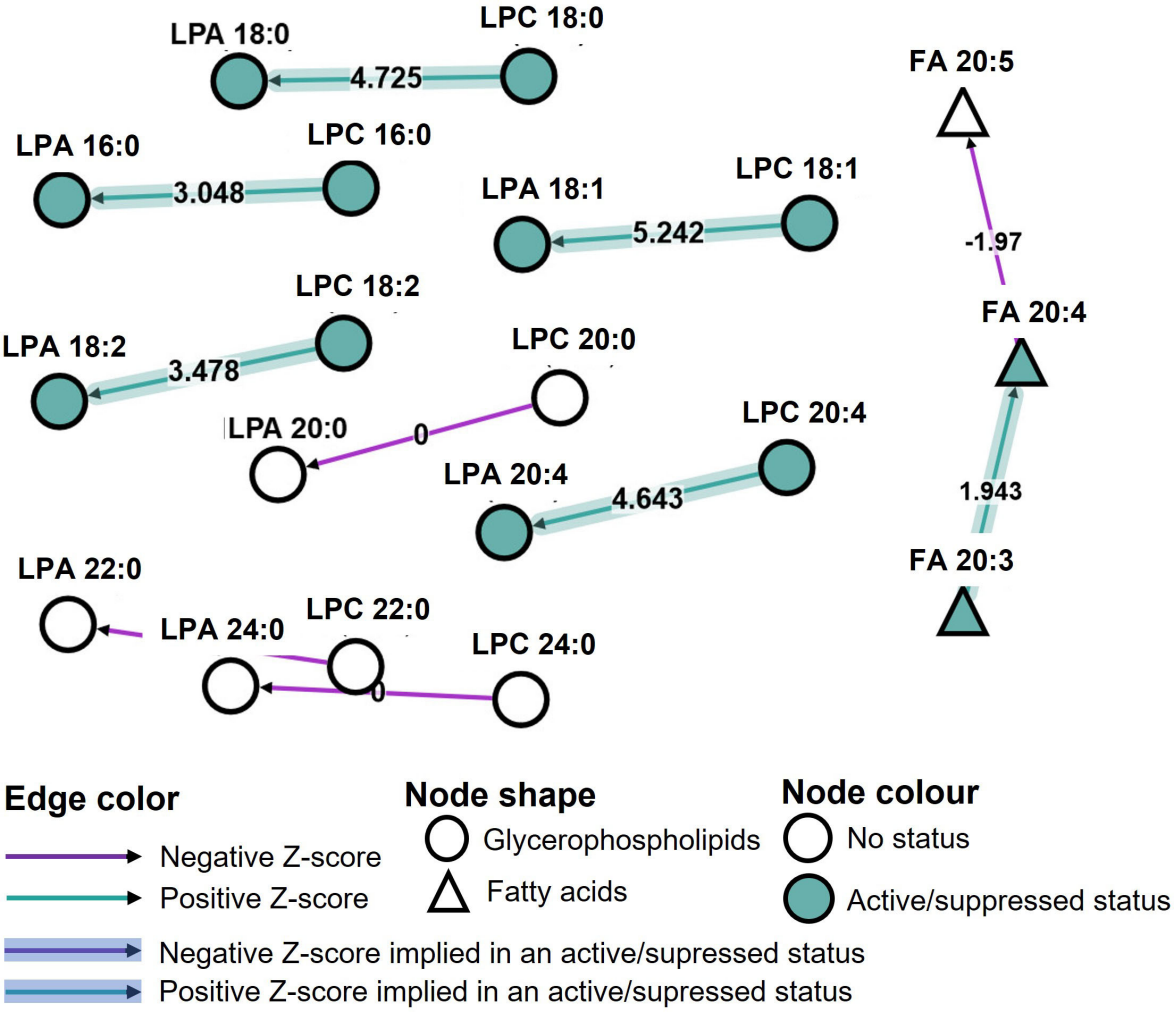
Pathway analysis of lipidomic data from Single Donor Apharesis during storage and in the event of adverse reactions. BioPan pathway analysis of lipidomic data from SDA-PC with and without occurrence of AR, regardless of storage time.

The storage time was not taken into consideration. SDA-PC without AR served as the control and the conditions analysed were SDA-PC with AR occurrence. The bioinformatic analysis mainly presented changes in LPC to LPA species with different z scores. LPA/C 20:0, 22:0 and 24:0 presented a null z score. In contrast, LPC 16:0; 18:0; 18:1; 18:2; 20:4 to LPA 16:0; 18:0; 18:1; 18:2; 20:4, respectively, are classed as active pathways (positive z score). FA 20:3 to FA 20:4 is also classified as active pathways (**Figure 6**). Interestingly, they are also classed as active pathways with an active status, which suggests that this is a prominent pathway in our data set (**Figure 6**). These bioinformatics analyses reinforce previous data (**Figures 1**–**5**). We then processed the data to compare SDA-PC with or without AR occurrence on days 1-2 of storage (**Supplementary Figure 8A**) and on days 3-4 of storage (**Supplementary Figure 8B**). We observed the same pathways and z score when we divided the analysis based on storage time. The bioinformatic analysis also revealed a pathway involving fatty acid. The software classified the following in FA 20:3: PGF_2_α and TXB_2_; in FA 20:4: 12-HETE, 15-HETE, 5-HETE, 8,9-EET, 8-HETE, LTB_4_, LXA_4_, PGA_1_, PGD_2_ and PGE_2_; in FA 20:5: 18-HEPE, 8isoPGA_2_, LTB_5._ Interestingly, a positive z score in active status was obtained between FA 20:3 and FA 20:4 and a negative z score between FA 20:4 and FA 20:5 (**Figure 6**). When we analysed storage time specifically, we noted the same positive or negative z score except that FA 20:3 to FA 20:4 is not expressed as an active status (**Supplementary Figures 8A, B**).

## Discussion

**Figure 7:**
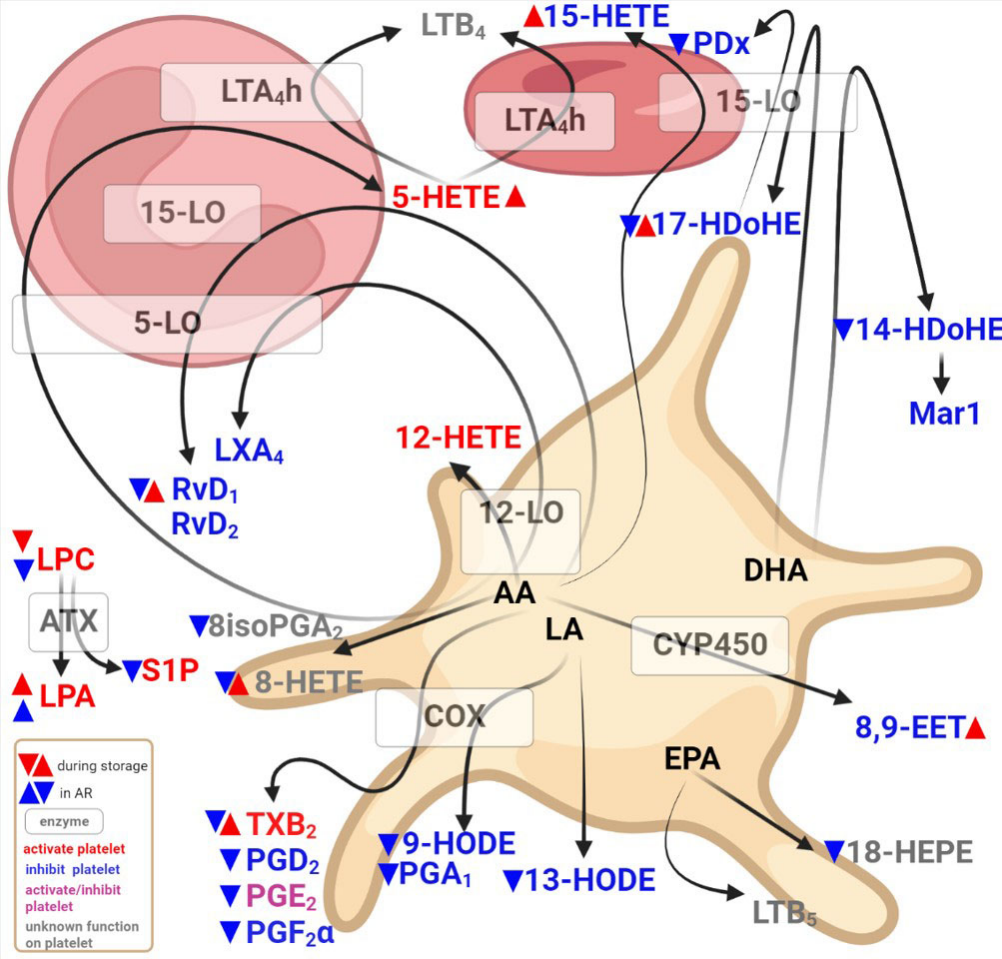
Diagram showing bioactive lipid detected in single donor apharesis during storage in the event of adverse reactions.

Other studies have assessed the lipid content of platelet concentrates (8, 25–27) or, in the case of a severe adverse reaction, a few lipids (28), but this is not combined with an extensive lipid mediator assessment or linked with an adverse reaction post-PC transfusion. Our study highlights bioactive lipids in SDA-PC during storage and some lipids that could shed light on the onset of adverse reaction in recipients (**Figure 7**). Indeed, in the case of an AR, LPA species expressions are increased in SDA-PC whereas other lipid mediator expressions are decreased. It is interesting to note that during SDA-PC storage, lipid concentrations rise for the LPA species, TXB_2_, 5-HETE, 8-HETE, 15-HETE, RvD_1_, RvD_2_, 17-HDoHE and 8,9-EET compared to LPC expressions, which decrease during storage (**Figure 7**).

The lipid content and the modulation thereof could be involved in the adverse reaction following SDA-PC transfusion. Lysophosphatidylcholine LPC was the main lysophospholipid studied in transfusion products. Even, the involvement of LPC in severe adverse reactions such as transfusion-related acute lung injury (TRALI) is still open to debate (27). LPC could prime polymorphonuclear cells, leading to endothelium adhesion and activation of the endothelium (29). This could explain the decrease in LPC species in the event of AR. However, other studies confirmed a significant increase in LPCs during storage (30). Interestingly, the differences in outcome between our study and others (30) could be explained by the variation in PC collection and processing, with particular reference to the use of additive solutions as described with other BRMs (31). Furthermore, polyunsaturated LPC species are deemed to be protective and prevent TRALI from occurring (32). Based on this finding, LPC 18:1 and 18:2 were found in copious quantities in SDA-PC without AR occurrence. However, their concentration decreased significantly in SDA-PC in the event of AR. LPC 18:1 and LPC 18:2 could be protective (**Supplementary Figures 1A, B**). We observed a decrease in LPC 18:1 and LPC 18:2 in SDA-PC prior to transfusion, and these SDA-PC lead to an adverse reaction. This could be due to their consumption by platelets and a decrease in the generation or catabolism of LPC species by phospholipase D or lysophospholipase such as autotaxin.

Bioinformatic analysis of our lipidomic data suggests the involvement of *PLD1* and/or *PLD2* in the process of PC to PA and LPC to LPA (**Figure 6** and **Supplementary Figure 8**). Phospholipase D (PLD) catalyses hydrolysis of the phospholipid diester bond to release phosphatidic acid and lipids. In humans, PLD enzymes are present intracellularly with PLD1 and PLD2 and may be secreted (autotaxin, ENPP2). The latter enzyme possesses lysoPLD activities. Lysophosphatidylcholine (LPC) and sphingosylphosphorylcholine (SPC) are autotaxin substrates. High expression of autotaxin are detected in the circulation and in an inflammatory context (21). Platelets constitute the major source of autotaxin in the circulation (33). Autotaxin expression is increased during day 3-4 of storage in SDA-PC with the occurrence of AR compared to SDA-PC without AR (**Figure 2B**). These observations confirm the inflammatory status of SDA-PC with the occurrence of AR. Indeed, autotaxin and their products such as LPA and S1P are involved and could be markers of future adverse reactions in transfusion. Inhibition of LPA should be tested during the storage of whole blood leukoreduction (34) and could be a predictor for sepsis with correlation of platelet count and vascular dysfunctions (35). On the contrary, the increasing presence of ATX in SDA-PC could affect the performance of the lipidomic analysis. Indeed, ATX could also increase the production of LPA species S1P, which could activate platelets (36). In turn, activated platelets could release a wide range of cytokines such as prostaglandin.

Our data corroborate the findings of studies focusing on PC storage and composition of platelet concentrates regarding EV and plasma in particular. These studies have reported increased platelet EVs with some LPA species (20). Other studies revealed a positive correlation between S1P and LPC but a negative correlation with ATX activity (37). LPA is well known for its activator role in relation to platelets, red blood cells and the endothelium (21, 38). Conversely, platelet activation increases lysophospholipid species such as LPC, lysophosphatidylethanolamine (LPE) and LPA (39). However, the involvement of LPA species in ARs such as TRALI is not well documented in the literature (33). LPA and LPC species contribute to the antithrombotic effect (40), displaying significant correlation with AR occurrence (**Supplementary Figure 4**). Indeed, our data show a decrease in S1P during PC storage and also in SDA-PC with AR occurrence (**Supplementary Figure 2**). Extracellular vesicles rich in S1P have a protective role. Higher EV levels were recorded in PCs stored over a 4-day period compared to PCs stored for 2 days. These EVs contained less S1P and mechanically induced endothelial cell barrier leakage. Supplementation of S1P or EV elimination prevents a severe adverse reaction (TRALI in this study) (41). Taken overall, an increase in LPA species, a decrease in LPC species, S1P and an increase in autotaxin could be strong predictors for an adverse reaction following platelet concentrate transfusion.

The SDA-PC lipid mediator content during storage was investigated previously (8, 42). However, the authors linked the lipid mediators with EV contained in the PC. Our study confirms the increase in prostaglandin and thromboxane during storage (**Supplementary Figure 6**). Nevertheless, in SDA-PC with AR occurrence, prostaglandin expressions decrease significantly suggesting that they are no longer produced and/or used to stimulate platelets in platelet concentrates. This observation suggests that prostaglandin generation was stopped or used for platelet stimulation prior to transfusion leading to AR. Platelets are sensitive to these prostaglandins. Indeed, PGD_2_ and PGF_2_α are platelet function inhibitors, whereas PGE_2_ inhibits or activates platelet function following activation by TXB_2_. The inhibition and activation of prostaglandin expression was balanced during storage. Indeed, platelets express various enzymes such as thromboxane synthase. They are also sensitive to these prostaglandins. PGD_2_ and PGF_2_α inhibit platelet functions whereas PGE_2_ inhibits/activates them. TXB_2,_ for its part, activates platelets. During storage, the inhibition and activation of prostaglandin expression was thus balanced (**Supplementary Figure 6**). The platelets did not express the necessary enzymes to produce di-ene and leukotriene. Platelets also expressed in their EVs, 12-lipoxygenase but not 5 and 15-lipoxygenase (14, 42). However, the lipidomic approach to our study reveals the presence of 5-HETE, 15-HETE, LTB_4_ and LXA_4_ in SDA-PC with or without AR occurrence. Their presence could contaminate the plasma. Indeed, the solution used to store PCs could contain at least 30% to 100% plasma with 0% to 70% of another solution, depending on the process (25).12-HETE activates platelets when LXA_4_ inhibits platelet functions (43, 44). Platelets have complex enzymatic machinery involving CYP (cytochrome p450) (23, 42). 8,9 EET detected in our study is derived from platelets. This lipid is known for its various effects such as vasodilation, anti-inflammation and anti-platelet aggregation. Lack of expression accompanied by prostaglandins in SDA-could potentially account for some AR symptoms.

Lipids derived from LA, DHA and EPA are also derived from platelets and another cell type such as leukocyte. PGA_1_, 9 and 13-HODE are generated from platelet LA contained in the plasma membrane. Their expression increases during storage of SDA-PC but decreases significantly in SDA-PC with the occurrence of AR. PGA_1_ is known as an inflammation regulator among other functions (45). 9-HODE increases adenylate cyclase in platelets as a partial agonist of PGE_1_ and PGE_2_ receptor. 13-HODE is mainly produced by endothelial cells; it inhibits TXB_2_ generation but increases 12-HETE generation (46) and inhibits platelet adhesion to the endothelium (47). These LA metabolites protect platelets against activation and aggregation (40). The decrease in the expression of these LA metabolites could be interesting in terms of diminished platelet activation but, in the transfusion context, platelet activation should be possible. LA metabolite expressions are low in SDA-PC with occurrence of AR. However, during the SDA-PC ageing process, these metabolite expressions are increased (**Supplementary Figure 6F**). These bioactive lipids could play a role in successful PC transfusion. Moreover, DHA metabolite expressions are also increased during SDA-PC storage and decrease in SDA-PC with AR occurrence. 14HDoHE could be generated by 12-lipoxygenase from platelets (48). Interestingly, PDx, RvD_1_, RvD_2_ prevent platelet aggregation, decrease inflammation by promoting the phagocytosis of blood clots by macrophages and prevent secondary thrombosis in the mice model (49–51). Our data confirmed the study conducted by Valkonen et al, which highlighted an increase in PC, PDx during storage and some resolving species such as RvD_1_, in platelet concentrate and in EVs isolated from PC (42). We were also able to detect 14-HDoHE (precursor of Maresin1) but not its product, Maresin1. Maresin1 interacts with platelets to enhance their haemostatic function and suppress their inflammatory role (43). One explanation could be that the maresin-1 contained in PC is completely used up by the platelet and therefore cannot be detected in the PC supernatant. Lastly, 18-HEPE and LTB_5_ are also detected during the storage of SDA-PC and their expression substantially decreases in SDA-PC leading to AR. These two bioactive lipids derived from EPA are considered inhibitors of platelet aggregation (40). Last but not least, the most abundant lipids detected are LPC and LPA species and 12-HETE>13-HODE>14-HDoHE. Their expressions are modulated through storage and decreased in SDA-PC leading to AR (**Supplementary Figures 2, 3, 6**). LPC, LPA and 12-HETE activate platelet functions whereas 13-HODE and 14-HDoHE are platelet inhibitors. The predominance of a lipid mediator activator in SDA-PC could account for the onset of adverse reactions following PC transfusion.

However, lipids are not the main force behind the onset of adverse reactions following platelet transfusion. The processes involved in PC generation (time, temperature) play a role in triggering adverse reactions (7, 25). It has been consistently shown that accumulations of Biological Response Modifiers (BRM) are responsible for adverse reactions (6). These reactions are triggered by biomolecules such as cytokines, lipids and also storage processes (time, temperature, etc.). During storage, the PC lipidome is affected by the composition of the platelet component (20, 25, 26, 52, 53), which could lead to TRALI in the mouse model (28). The platelet storage lesion induced platelet activation, decreased the quality of the PC and led to platelet clearance post-transfusion (54). Proteomic analysis highlights some changes in PC components such as CD40L, PDGF and cytokines (IL1β, IL6, IL27) involved in adverse reaction occurrence (7, 25, 54). Moreover, the state of the patient who received the platelet concentrate transfusion is very important to consider. Indeed, some patient received several blood products, have comorbidities and severe disease. It is one of limit of our study, as we did not have access to clinical date regarding patient histories.

In conclusion, this study assessed lipid mediators in single donor apharesis platelet concentrates during storage and in the onset of adverse reactions post-transfusion. We demonstrated modulation of bioactive lipids during storage, mainly in the form of the platelet function activator. However, all lipids, except LPA species, are low in PC with AR occurrence compared to PC without AR occurrence. Based on these data and with bioinformatic analysis, we suggest that the imbalance between LPA and LPC expressions could be a marker for AR occurrence and could be targeted in the future to enhance PC transfusion (**Figure 7**).

## Data availability statement

The raw data supporting the conclusions of this article will be made available by the authors, without undue reservation.

## Author contributions

FC, OG and HH-C designed the study, supervised the research, secured funding and obtained approval from the ethics committee. A-CD analysed data. A-CD wrote the manuscript. A-CD and FC reviewed the manuscript. SF-D, CS, TE, MH, C-AA, M-AE, AP, EA, JB-M, BP, EB conducted research. All authors contributed to the article and approved the submitted version.
