# Supplementary material for: Bioactive lipids as biomarkers of adverse reactions associated with apheresis platelet concentrate transfusion
Source: Front Immunol. 2023 Apr 17;14:1031968. doi: 10.3389/fimmu.2023.1031968 (PMC10149858; doi:10.3389/fimmu.2023.1031968)
Supplement: Supplementary file 1 [file DataSheet_1.pdf]

Supplementary information

Material and Methods

Ethic Statement

Single Donor Apheresis - Platelet Concentrates (SDA-PC) were obtained from “Etablissement Français du Sang (EFS) Auvergne-Rhone-Alpes” with 9,206 volunteers enrolled between March 2013 and February 2016 giving their informed consent. The study was approved by EFS‘s institutional review board for ethics (DC-2019-3803 & AC-2020-3959). Only 2,850 PCs were sampled. 140 Adverse Reactions (AR) were reported upon transfusion and were investigated in a previous study.(1) The residual SDA-PCs transfused were collected. Lipidomic analysis was performed on 33 SDA-PC AR. We randomly selected 25 SDA-PC reported without AR, as controls.

Mass spectrometry

Liquid chromatography tandem mass spectrometry (LC-MS/MS) analysis was performed by the MetaToul-Lipidomic MetaboHUB Core Facility, France.

Oxylipin extraction and measurement

100 µL of plasma were withdrawn for oxylipins analyses. 300 µL of cold methanol and 40 µL of internal standard (Deuterium labeled compounds) were added. After centrifugation at 5000 g for 15 min at 4°C, supernatants were transferred into 2 mL 96-well deep plates and diluted in H_2O to 2 mL. Samples were then submitted to solid phase extraction (SPE) using OASIS HLB 96-well plate (30 mg/well, Waters) pretreated with MeOH (1mL) and equilibrated with 10% MeOH (1 mL). After sample application, extraction plate was washed with 10% MeOH (1 mL). After drying under aspiration, lipids mediators were eluted with 1 mL of MeOH. Prior to LC-MS/MS analysis, samples were evaporated under nitrogen gas and reconstituted in 10 µL on MeOH.

LC-MS/MS analyses of eicosanoids were performed as described.(2) Briefly, lipid mediators were separated on a ZorBAX SB-C18 column (2.1 mm, 100 mm, 1.8 µm) (Agilent Technologies) using Agilent 1290 Infinity HPLC system (Technologies) coupled to an ESI-triple quadruple G6460 mass spectrometer (Agilent Technologies). Data were acquired in Multiple Reaction Monitoring (MRM) mode with optimized conditions (ion optics and collision energy). Peak detection, integration and quantitative analysis were done using Mass Hunter Quantitative analysis software (Agilent Technologies) based on calibration lines built with commercially available eicosanoids standards (Cayman Chemicals).

Lysophospholipid extraction and measurement

Sample (25 µL) were adjusted to 500 µL with deionized water before the addition of 500 µL non-acidified 1-butanol containing 50 ng of internal standard LPA 17:0 and 10 ng of internal standard LPC 17 :0. The samples were vortexed every 30 minutes for 2 h before centrifugation at 10 000 g for 20 minutes. The upper organic phase was transferred to a new tube and evaporated to dryness under reduced pressure using a Rotavapor. The dried lipids were dissolved in 160 µL MeOH then filtrated on a 0.45 µm polyterafluoroethylene membrane filter. The eluate is evaporated and dissolved in 10 µL MeOH. The extract was then stored at–20 °C before LC-MS/MS analysis.
Calibration curve. A mixture of LPC and LPA species (calibration solution) was prepared in MeOH (Vf 50µl) at 1000 ng/mL to obtain ten calibration points (dilution by half) with fixe-derived internal standard concentration [LPC17] = 100 ng/mL, and [LPA17] = 500 ng/mL. Calibration curves were calculated by the IS method using the area ratio between the analyte and the internal standard.

Liquid chromatography mass spectrometry. High-performance liquid chromatography was performed using an Agilent 1290 Infinity (Agilent Technologies) equipped with an auto sampler, a binary pump and a column oven.

The analytical column was an Acquity UPLC BEH-C8 (100 x 2,1 mm, 1,7 µm) (Waters) maintained at 25 °C. The mobile phases consisted of Water, FA and MeOH (20:0,5:79,5; v/v/v) (A) and MeOH, FA (99,5:0,5, v/v) (B). The two mobile phases contained 5 mM ammonium formate. The gradient was as follows: 0% B at 0 min, 0% B at 1 min, 100% B at 2 min, 100% B at 5 min, 0% B at 6 min and 0% B at 8 min. The flow rate was 0,2 mL/min. The auto sampler was set at 5 °C and the injection volume was 5 µL. The HPLC system was coupled on-line to an Agilent 6460 triple quadrupole MS (Agilent Technologies) equipped with electrospray ionization source. Electrospray ionization (ESI) was performed in positive ion mode for LPC and negative ion mode for LPA. So two acquisitions are necessary. After optimization, the source parameters used were as follows: source temperature was set at 300°C, nebulizer gas (nitrogen) flow rate was 10 L/min, sheath gas temperature was 300°C, sheath gas (nitrogen) flow rate was 12 L/min and the spray voltage was adjusted to +4000 V. The collision energy optimums for LPA species were 20 eV (except LPA 20:0 EC=22 eV). The collision energy for LPC species were 30 eV (except LPC 22:0 EC=35 eV). Analyses were performed in Selected Reaction Monitoring detection mode (SRM) using nitrogen as collision gas. Finally, peak detection, integration and quantitative analysis were done using MassHunter QqQ Quantitative analysis software (Agilent Technologies) and Microsoft Excel software.

Acknowledgments

The authors wish to thank the blood donors who contributed samples for the study. The authors are grateful to our Masters student for contributing original data. We would also like to thank the medical staff at Etablissement Français du Sang Auvergne-Rhone-Alpes, Saint-Etienne, France for their technical support throughout this study. This work was supported by grants from Etablissement Français du Sang (EFS) – the French National Blood Establishment - and the Association “Les Amis de Rémi” Savigneux, France.

Authorship

Contribution: F.C., O.G, E.B and H.H.C designed the study, supervised the research, secured funding and obtained approval from the ethics committee; A.C.D analysed data. A.C.D wrote the manuscript. A.C.D and F.C reviewed the manuscript. S.F.D, C.S, T.E, C.A.A, M.A.M, A.P, E.A, J.C.P, J.B.M and B.P conducted research.

References

1. Cognasse F, Sut C, Fromont E, Laradi S, Hamzeh-Cognasse H, Garraud O. Platelet soluble CD40-ligand level is associated with transfusion adverse reactions in a mixed threshold-and-hit model. Blood. 2017;130(11):1380-3.
2. Le Faouder P, Baillif V, Spreadbury I, Motta JP, Rousset P, Chene G, et al. LC-MS/MS method for rapid and concomitant quantification of pro-inflammatory and pro-resolving polyunsaturated fatty acid metabolites. J Chromatogr B Analyt Technol Biomed Life Sci. 2013;932:123-33.

Supplementary Table 1: Lipid mediator assessed in single donor apheresis platelet concentrate with or without adverse reaction

Eicosanoids	Detection
LPC 16:0	detected
LPC 18:0	detected
LPC 18:1	detected
LPC 18:2	detected
LPC 20:0	detected
LPC 20:4	detected
LPC 22:0	detected
LPC 24:0	detected
LPA 16:0	detected
LPA 18:0	detected
LPA 18:1	detected
LPA 18:2	detected
LPA 20:0	detected
LPA 20:4	detected
LPA 22:0	detected
LPA 24:0	detected
S1P 18:1	detected
PGA_1	detected
8iso-PGA_2	detected
PGD_2	detected
PGE_2	detected
PGE_3	non detected
$PGF_2\alpha$	detected
11β-$PGF_2\alpha$	non detected
6keto-$PGF_1\alpha$	non detected
15dPGJ_2	non detected
TXB_2	detected
5-HETE	detected
5oxoETE	non detected
5,6-DiHETE	non detected
8-HETE	detected
12-HETE	detected
15-HETE	detected
LTB_4	detected
LTB_5	detected
LXA_4	detected
LxB4	non detected
RvD_1	detected
RvD_2	detected
7MaR1	non detected
PDx	detected
5,6-EET	non detected
8,9-EET	detected
11,12-EET	non detected
14,15-EET	non detected
9-HODE	detected
13-HODE	detected
14-HDoHE	detected
17-HDoHE	detected
18-HEPE	detected

Supplementary Figures and legends
Supplementary Figure 1

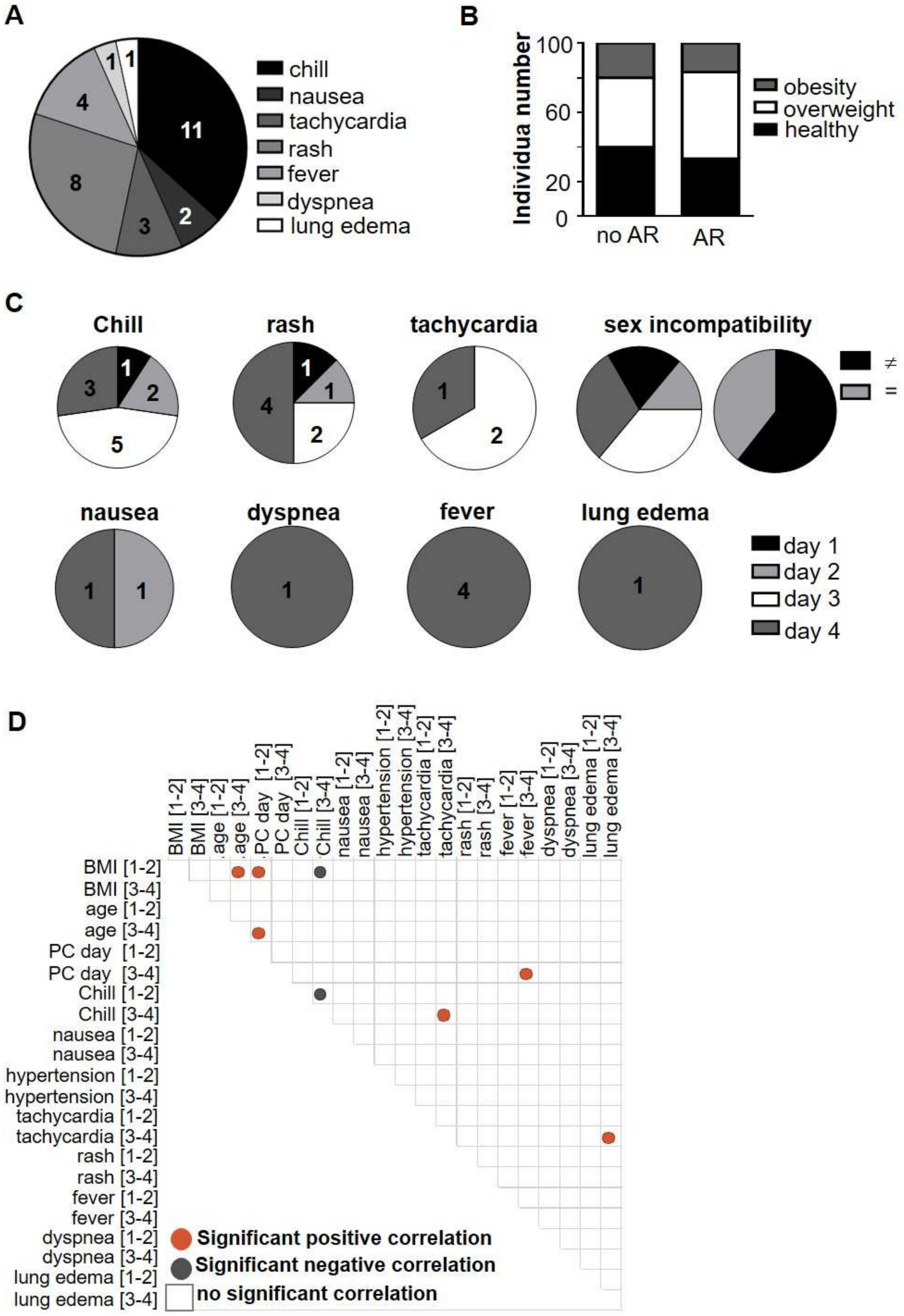

Supplementary Figure 1. Symptoms of adverse reaction and correlation with clinical information

(A) Pie Chart representing the proportion of symptoms of adverse reactions during our study (n=36 SDA-PC with occurrence of AR). 6 SDA-PC reported with AR are not linked with the symptom mentioned. The number reported in each part are the raw number of patient who suffered of adverse reaction symptom following a transfusion. (B) Graph representing the proportion of obese versus healthy BMI donors of SDA without AR (n=29) and with AR (n=36). (C) Pie chart representing different adverse reactions during the storage time. (D) Correlation matrix with clinical information and symptoms of adverse reactions. Correlation matrices are built with a Pearson correlation. Grey dots represent a negative correlation and red dots a positive correlation, with the level of significance being $p < 0.05$.

Supplementary Figure 2

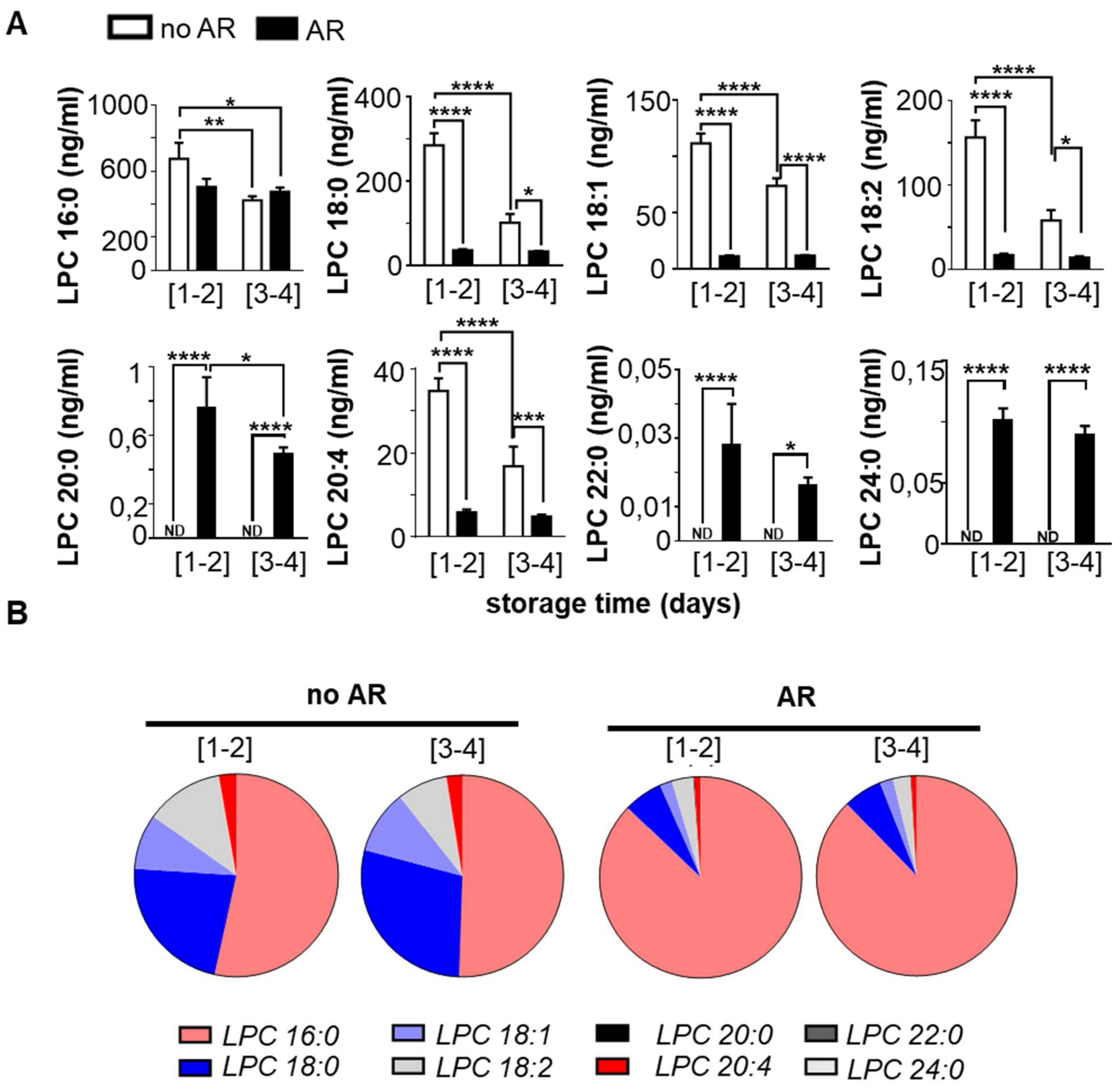

Supplementary Figure 2. Comparison of lysophosphatidylcholine profile between Single Donor Apharesis with or without adverse reaction occurrence.

(A) LPC generation in Single Donor Apheresis (SDA-PC) with or without AR occurrence during storage. The white bar represents the mean ± SEM of LPC species concentration in SDA-PC without occurrence of AR and with occurrence of AR (black bar). (To refer to n patient, see Table 1). Statistical analysis performed with 2-way ANOVA *p<0.05; **p<0.005; ***p<0.0005, ****p<0.0001. (B) Pie chart representing the proportion of LPC species in SDA-PC with or without AR occurrence.

Supplementary Figure 3

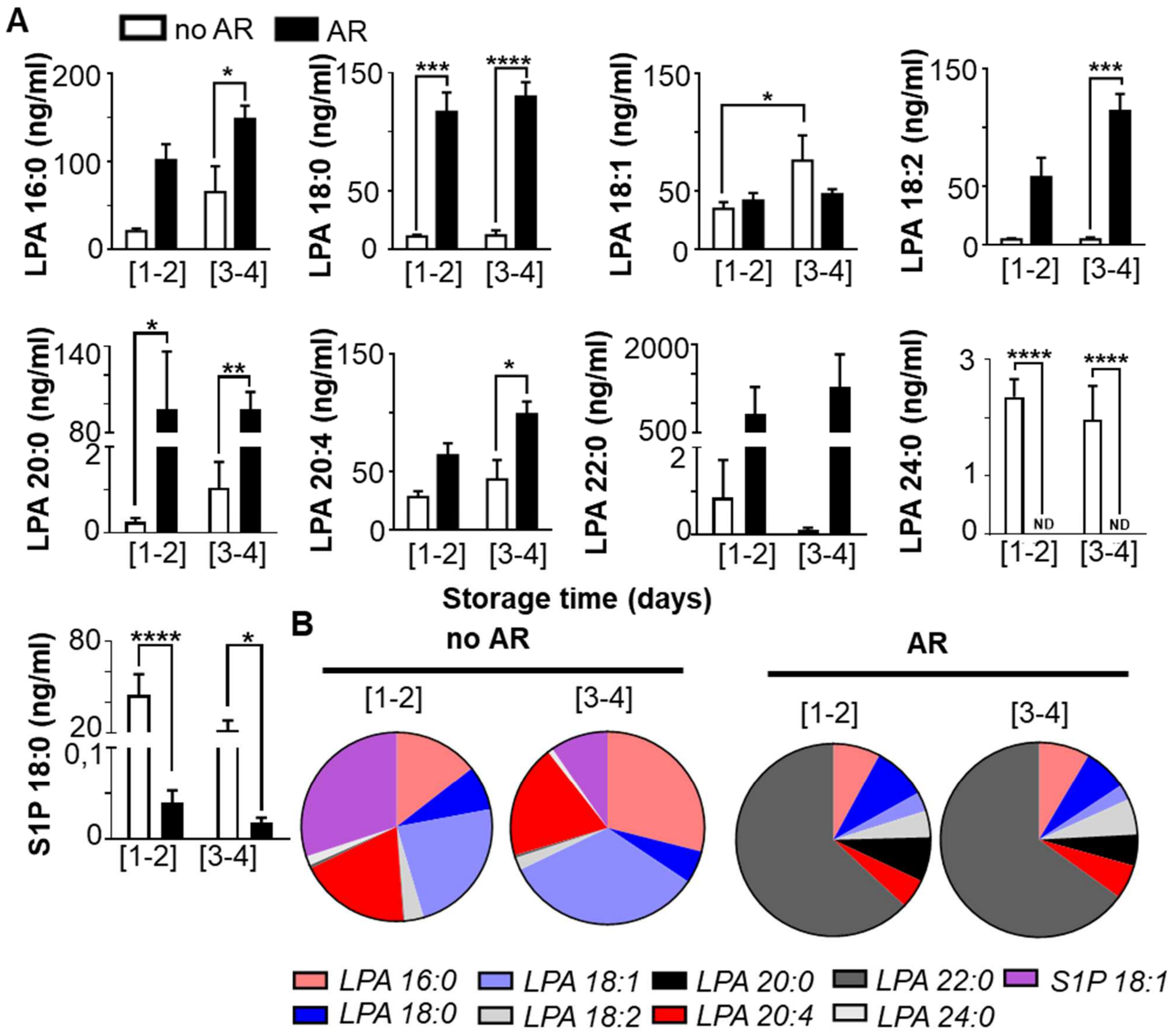

Supplementary Figure 3. Correlative study of autotaxin product profile between Single Donor Apheresis with or without an adverse reaction.

(A) LPA and S1P generation in Single Donor Apheresis (SDA-PC) with or without AR occurrence during storage. White bar represents the mean $\pm$ SEM of bioactive lipid concentration in SDA-PC without occurrence of AR and with occurrence of AR (black bar). (To refer to n patient, see Table 1). Statistical analysis performed with 2-way ANOVA *$p<0.05$; **$p<0.005$; ***$p<0.0005$, ****$p<0.0001$. (B) The pie chart represents the proportion of LPA and S1P species in SDA-PC with or without AR occurrence.

Supplementary Figure 4

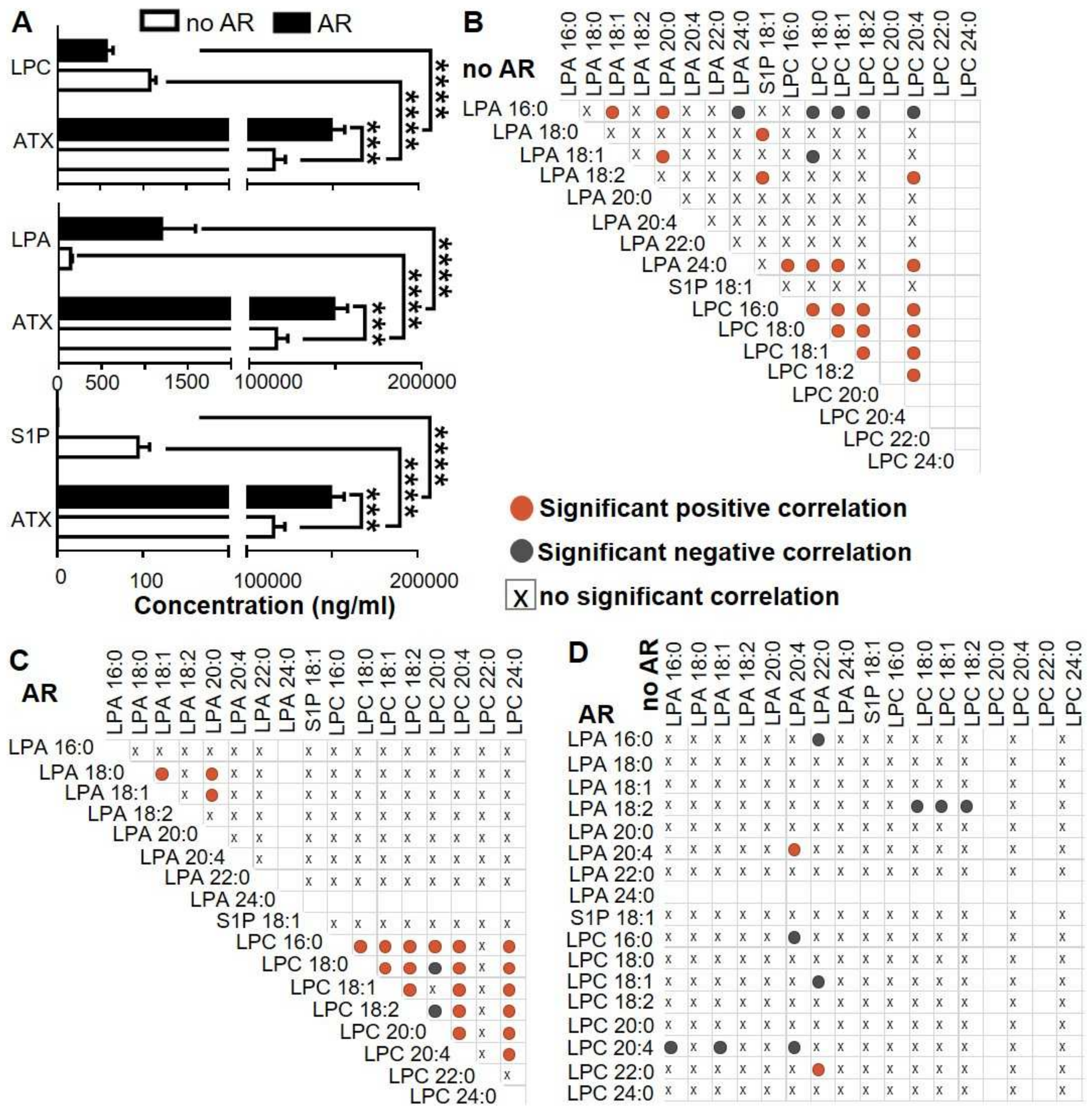

Supplementary Figure 4. Correlation between adverse reaction symptoms and lipidomic analysis

(A) Graph bar represents the mean ± SEM of total LPC, LPA or S1P concentration with ATX expression at storage time 0 in SDA-PC with or without AR occurrence. The white bar represents the lipid concentration in SDA-PC without AR occurrence and the black bar denotes SDA-PC with AR occurrence. (n= 5 times). Statistical analysis performed with 2-way ANOVA *p<0.05; **p<0.005; ***p<0.0005, ****p<0.0001. (B) Correlation matrix of S1P, LPA and LPC species in SDA-PC without AR occurrence, taking all storage times into account. (C) Correlation matrix of S1P, LPA and LPC species in SDA-PC with occurrence of AR, taking all storage times into account. (D) Correlation matrix of S1P, LPA and LPC species in SDA-PC with occurrence of AR compared to SDA-PC without occurrence of AR. Correlation matrices are built with Pearson correlation. The grey dots represent a negative correlation and red dots a positive correlation, with the level of significance being p <0.05.

Supplementary Figure 5

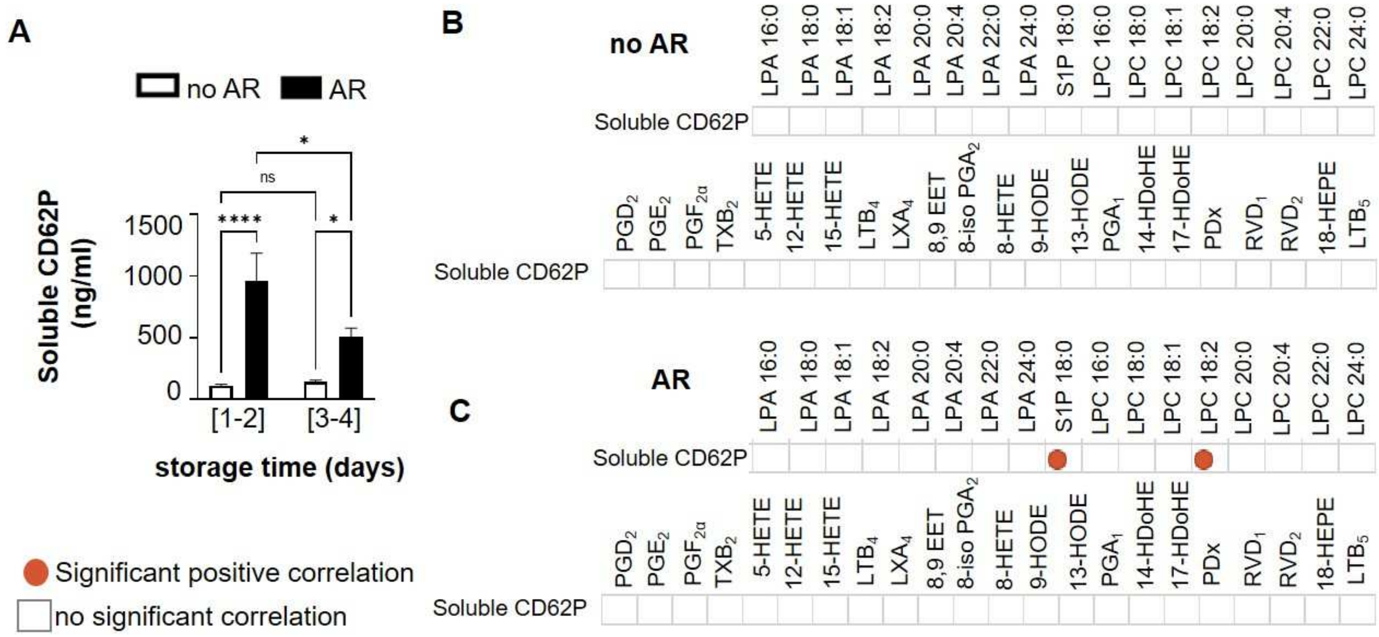

Supplementary Figure 5. platelet activation status and correlation matrix between lipid and activation status.

(A) The graph bar represent the mean ± SEM of soluble CD62P expression in SDA-PC with or without occurrence of AR. Statistical analysis performed with 2-way ANOVA *p<0.05; ****p<0.0001. (B) Pearson correlation matrix between lysophospholipid and p-selectin. (C) Pearson correlation matrix between lipid mediators and p-selectin. Red dots represent a positive correlation, with the level of significance being p <0.05.

Supplementary Figure 6

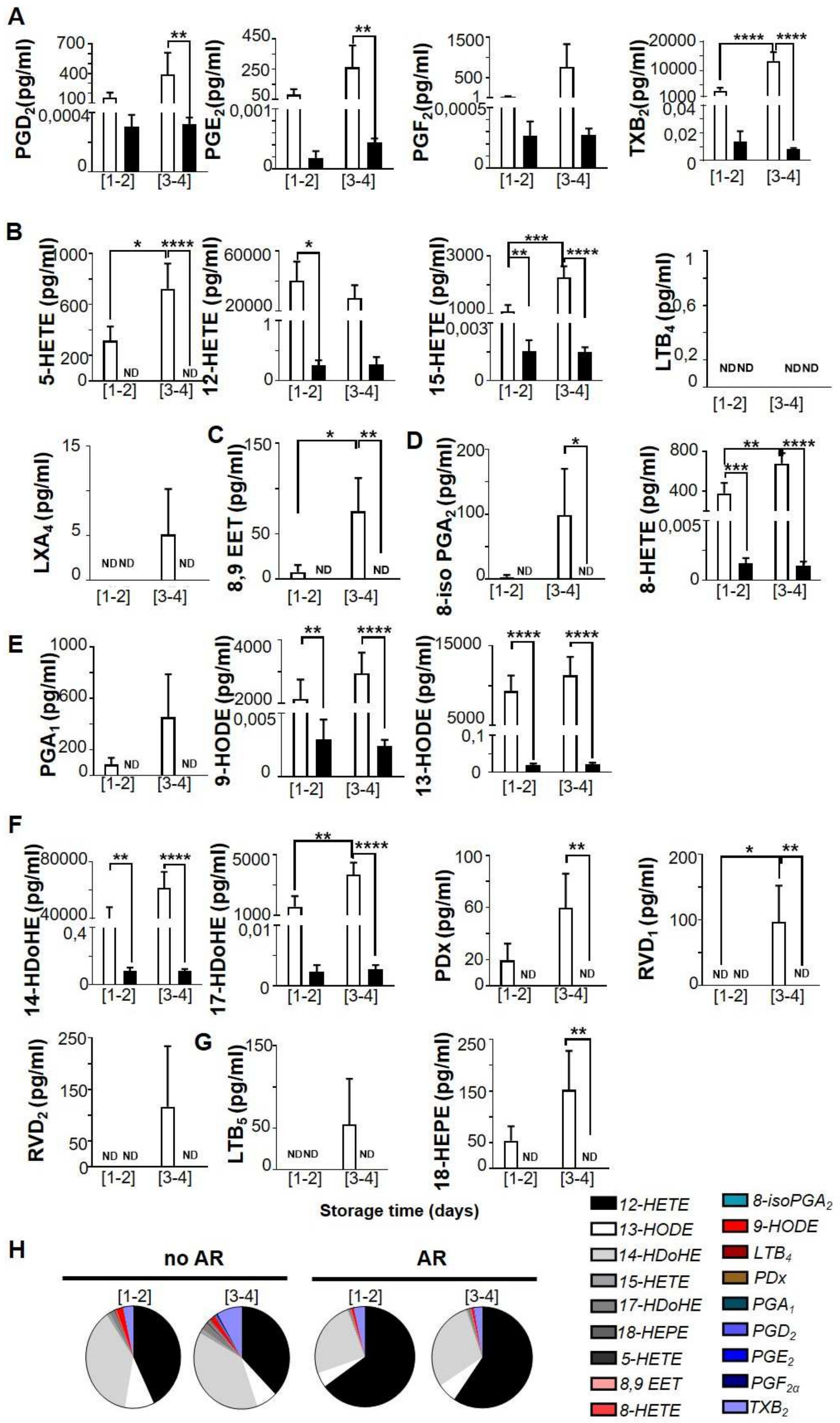

Supplementary Figure 6. Comparison of lipid mediator profile between Single Donor Apharesis with or without adverse reaction occurrence.

The graph bars represent mean + SEM of lipid mediator concentration. (A) COX products from AA generation during SDA with or without AR (To refer to n patient, see Table 1). Statistical analysis performed with 2-way ANOVA *p<0.05; **p<0.005; ***p<0.0005, ****p<0.0001. (B) LO products from AA generation during SDA with or without AR occurrence (To refer to n patient, see Table 1). Statistical analysis performed with 2-way ANOVA *p<0.05; **p<0.005; ***p<0.0005, ****p<0.0001. (C) CYP-450 products from AA generation during SDA-PC with or without AR occurrence (To refer to n patient, see Table 1). Statistical analysis performed with 2-way ANOVA *p<0.05; **p<0.005; ***p<0.0005, ****p<0.0001. (D) non-enzymatic products from AA generation during SDA-PC with or without AR (To refer to n patient, see Table 1). Statistical analysis performed with 2-way ANOVA *p<0.05; **p<0.005; ***p<0.0005, ****p<0.0001. (E) Products from LA generation during SDA-PC with or without AR occurrence (To refer to n patient, see Table 1). Statistical analysis performed with 2-way ANOVA *p<0.05; **p<0.005; ***p<0.0005, ****p<0.0001. (F) ¨Products from DHA generation during SDA-PC with or without AR occurrence (To refer to n patient, see Table 1). Statistical analysis performed with 2-way ANOVA *p<0.05; **p<0.005; ***p<0.0005, ****p<0.0001. (G) Products from EPA generation during SDA-PC with or without AR occurrence (To refer to n patient, see Table 1). Statistical analysis performed with 2-way ANOVA *p<0.05; **p<0.005; ***p<0.0005, ****p<0.0001. (H) Pie chart representing the proportion of eicosanoid in SDA-PC with or without AR occurrence.

Supplementary Figure 7

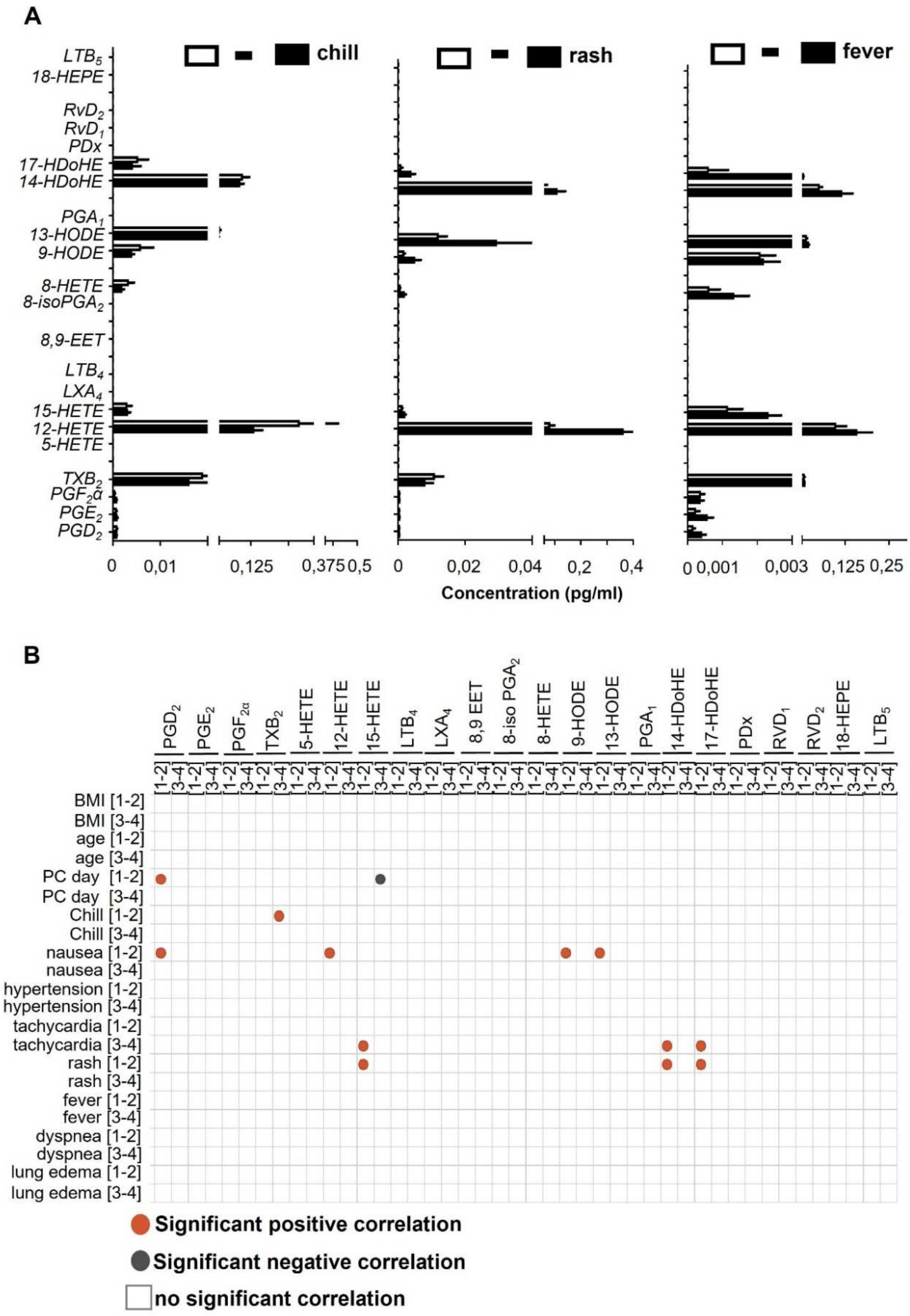

Supplementary Figure 7. Correlation between bioactive lipids and adverse reaction symptoms. (A) Graph bar represent the mean ± SEM of lipidomic data of SDA-PC with occurrence of AR for chill (n=11), rash (n= 8) or fever (n=4). All data are from SDA-PC with AR occurrence. The white bar represents expression of bioactive lipids without symptoms and the black bar denotes a symptom reported on the graph. (B) Correlation matrix between bioactive lipids and adverse reaction symptoms is built with Pearson correlation. Grey dots represent a negative correlation and red dots a positive correlation, with the level of significance being $p < 0.05$.

Supplementary Figure 8

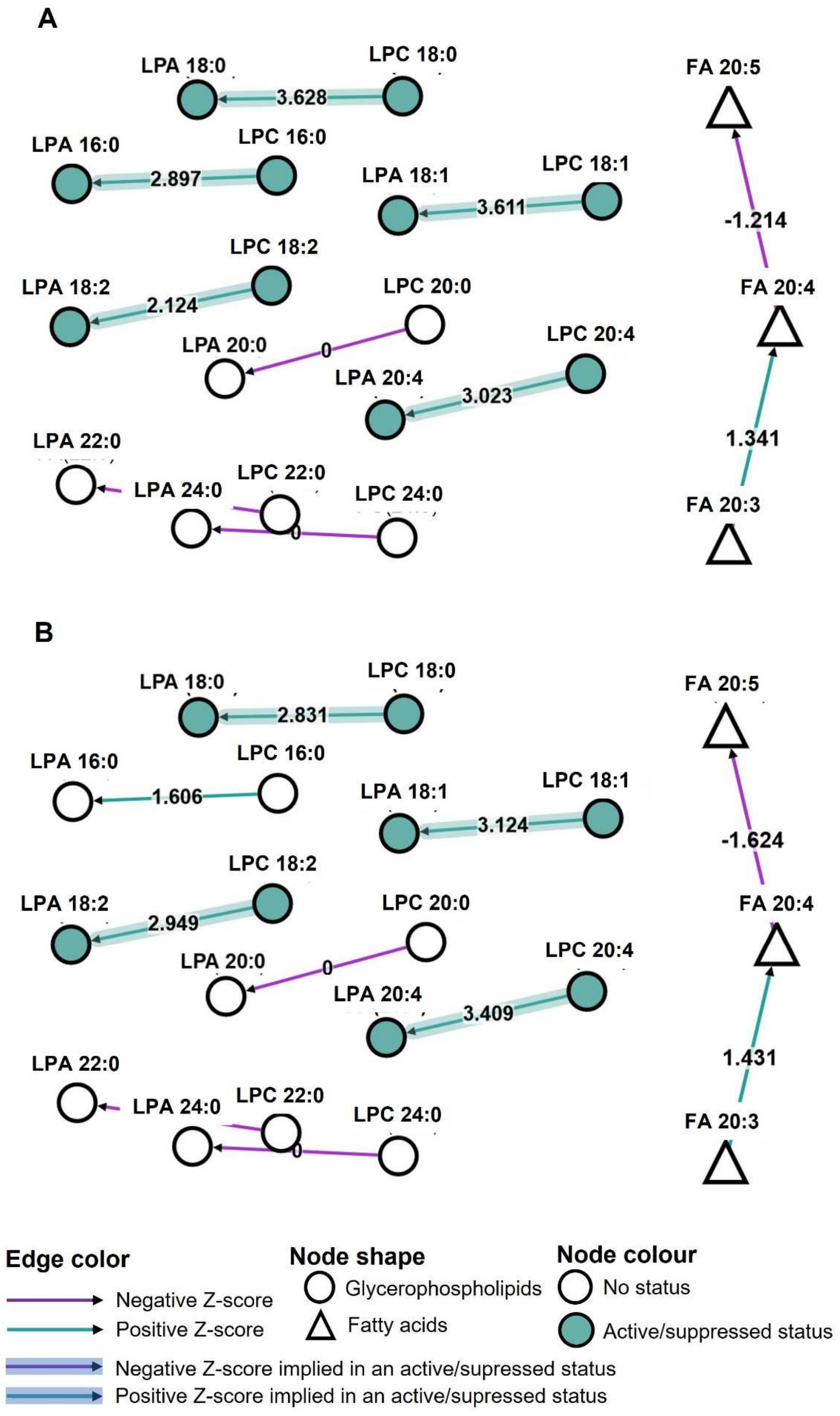

Supplementary Figure 8. Pathway analysis of lipidomic data from Single Donor Apheresis during storage and with the occurrence of adverse reactions.
BioPan pathway analysis of lipidomic data from SDA-PC with and without occurrence of AR depending on storage time: day [1-2] (A), day [3-4] (B).
